# Disentangling the Complexity of HGF Signaling by Combining Qualitative and Quantitative Modeling

**DOI:** 10.1371/journal.pcbi.1004192

**Published:** 2015-04-23

**Authors:** Lorenza A. D’Alessandro, Regina Samaga, Tim Maiwald, Seong-Hwan Rho, Sandra Bonefas, Andreas Raue, Nao Iwamoto, Alexandra Kienast, Katharina Waldow, Rene Meyer, Marcel Schilling, Jens Timmer, Steffen Klamt, Ursula Klingmüller

**Affiliations:** 1 Division Systems Biology of Signal Transduction, German Cancer Research Center (DKFZ), INF 280, Heidelberg, Germany; 2 Max Planck Institute for Dynamics of Complex Technical Systems, Magdeburg, Germany; 3 Institute of Physics, University of Freiburg, Freiburg, Germany; 4 BIOSS Centre for Biological Signalling Studies, University of Freiburg, Freiburg, Germany; 5 Merrimack Pharmaceuticals, Inc., Cambridge, Massachusetts, United States of America; Northeastern University, UNITED STATES

## Abstract

Signaling pathways are characterized by crosstalk, feedback and feedforward mechanisms giving rise to highly complex and cell-context specific signaling networks. Dissecting the underlying relations is crucial to predict the impact of targeted perturbations. However, a major challenge in identifying cell-context specific signaling networks is the enormous number of potentially possible interactions. Here, we report a novel hybrid mathematical modeling strategy to systematically unravel hepatocyte growth factor (HGF) stimulated phosphoinositide-3-kinase (PI3K) and mitogen activated protein kinase (MAPK) signaling, which critically contribute to liver regeneration. By combining time-resolved quantitative experimental data generated in primary mouse hepatocytes with interaction graph and ordinary differential equation modeling, we identify and experimentally validate a network structure that represents the experimental data best and indicates specific crosstalk mechanisms. Whereas the identified network is robust against single perturbations, combinatorial inhibition strategies are predicted that result in strong reduction of Akt and ERK activation. Thus, by capitalizing on the advantages of the two modeling approaches, we reduce the high combinatorial complexity and identify cell-context specific signaling networks.

## Author Summary

Cellular responses to extracellular stimuli are driven by activation of intracellular signaling pathways. The interconnections between signaling pathways contribute to the high complexity of signaling networks, therefore playing an important role in response to treatment in pathological conditions. Thus, unraveling the network structure in a cell-context specific manner is key to predict cellular responses to perturbations. Here, we present a novel hybrid mathematical modeling strategy taking advantage of qualitative and quantitative modeling approaches. We combine interaction graph and dynamic modeling with quantitative experimental data to study the hepatocyte growth factor induced signaling network in primary mouse hepatocytes. Specifically, we analyze the interconnections within and between PI3K and MAPK signaling pathways involved in hepatocytes proliferation. Based on literature knowledge, more than 100000 potential network structures are possible. By applying our approach, we reduce this combinatorial complexity and select 16 minimal model structures. Subsequently, by performing a systematic model selection we select the model structure representing the experimental data best. We experimentally validate the resulting best model structure, and, based on model simulation, we are able to predict the outcome of combinatorial treatments. Our hybrid approach is applied to unravel cell-context specific network structures and to predict the outcome of intervention strategies.

## Introduction

Cells receive extracellular signals and process them through intracellular signaling pathways to regulate cellular responses. Traditionally, signaling cascades were interpreted as linear chains of events. However, signaling pathways involve extensive crosstalk and feedforward as well as feedback loops resulting in complex, non-linear intracellular signaling networks, whose topologies are often context-specific and altered in diseases [1].

An important factor that contributes to liver regeneration and has been implicated in the context of resistance to targeted tumor therapy is hepatocyte growth factor (HGF). HGF is the key growth and survival factor for hepatocytes [2, 3] and in response to liver damage facilitates restoration of the tissue mass by promoting proliferation of hepatocytes. Upon binding to its receptor Met, HGF activates the phosphoinositide-(PI)-3-kinase (PI3K) and the mitogen activated protein kinase (MAPK) signaling pathways. Conditional knock-in mice that harbor an inactivating mutation of Met show reduced activation of PI3K signaling and complete abrogation of the activation of the MAPK pathway in response to partial hepatectomy [2]. As a consequence, damage repair is impaired in these mice suggesting an important role for the crosstalk of the signaling pathways. Therefore, to gain insights into the mechanisms controlling hepatocyte proliferation during liver regeneration, it is important to unravel mechanisms of feedback and crosstalk regulation that are relevant in hepatocytes.

In general, activation of PI3K leads to the generation of phosphatidylinositol 3,4,5–triphosphate (PI3,4,5-P_3_) that serves as docking site for the serine/threonine protein kinase Akt at the plasma membrane. Akt is activated by phosphorylation on serine 473 and threonine 308 and subsequently phosphorylates multiple substrates with important functions in key biological responses. While PI3K can be activated by direct binding to the receptor, MAPK signaling requires the activation of SOS and Ras that in turn activates Raf initiating the MAPK signaling cascade. Activated Raf leads to phosphorylation of a dual specific kinase, the mitogen-activated protein kinase kinase (MEK1 and 2), that phosphorylates the extracellular-signal regulated kinase (ERK1 and 2). Dual phosphorylated ERK regulates cytoplasmic and nuclear factors and thereby modulates numerous biological responses such as proliferation, differentiation and survival. Both signaling pathways are tightly interlinked and several mechanisms have been proposed for feedback loops within and crosstalk between PI3K and MAPK signaling (S2–S3 Tables). For example, it was shown that within the MAPK signaling pathway a negative feedback loop is triggered by ERK or p90RSK targeting SOS [4], and a positive feedback loop operates from ERK to Raf via RKIP, a protein playing a dual role as positive and negative regulator of MAPK signaling [5]. Furthermore, a positive feedback loop enhancing Gab1 activation via PI3,4,5-P_3_ generation was identified within the HGF induced PI3K signaling pathway [6]. Upon IGF induced stimulation, a negative interaction between PI3K and MAPK signaling pathways was reported, as Akt mediated phosphorylation of Raf on serine 259 leads to inactivation of Raf [7]. The majority of studies identifying these mechanisms were performed with tumor cell lines that harbor key alterations in signaling pathways controlling, for example, cell proliferation. Therefore, studies with primary cells are essential to identify physiologically relevant mechanisms. Furthermore, if we assume the 17 most likely crosstalk and feedback mechanisms between PI3K and MAPK signaling pathways (S3 Table), 131072 (2^17^) possible network structures are conceivable. Thus, due to the high combinatorial complexity, a systematic method is required to facilitate unbiased identification of cell-context specific structure of signaling networks.

Knowledge of cell-context specific feedback and crosstalk mechanisms is central to gain insights into mechanisms that cause undesired effects of targeted therapy. Several compounds targeting individual components in PI3K and MAPK signaling have been developed, and multiple clinical trials were initiated [8–10]. Inhibitors targeting the PI3K signaling pathway include the reversible PI3K inhibitor LY29004, the more potent irreversible inhibitor Wortmannin and the allosteric Akt inhibitor VIII. Derivatives of Wortmannin, such as PX-866, other PI3K inhibitors such as XL-147 [11] and CAL-101 [12] as well as the allosteric pan-Akt inhibitor MK-2206 [13, 14] are currently used in clinical trials. To analyze the MAPK signaling pathway, the compounds PD 98059 and U0126 inhibiting MEK have been widely applied. Several MEK inhibitors entered the clinical trials including, for example, CI-1040 and its analogue PD 0325901 [15–18]. Despite the specificity of these inhibitors, their efficacy was reported to be very limited. Combinatorial treatment with the MEK inhibitor ARRAY-438162 in combination with the PI3K inhibitors BYL719 and BKM120 or the Raf inhibitors LGX818 and RAF265 are ongoing [19, 20]. Furthermore, BX-912 is the PDK1 inhibitor that is primarily used for the analysis of this signaling pathway, whereas the PDK1 inhibitor AR-12 was tested in a clinical trial and showed limited absorption as well as pharmacokinetic variability [21]. Although multiple compounds are available, strategies to improve their application and to select most promising combinations remain to be developed.

To this aim, mathematical models provide unique tools to disentangle complexity and to predict the impact of perturbations. Mathematical models of the MAPK signaling pathway have been developed that only consider negative feedback [22], negative and positive feedback loops [5] or that analyze the signal-to-response relation [23]. Mathematical models describing both PI3K and MAPK signaling pathways upon single or combinatorial stimuli reveal the presence of crosstalk mechanisms between MAPK and PI3K pathways [24–26] or differences in the stimulus specific network topology [27, 28]. As indicated, most of the studies considered only single feedbacks or a limited number of crosstalk mechanisms. Therefore, to unravel a more complex network structure, a systematic unbiased approach is required.

Several computational methods have been developed to infer and analyze signaling networks. Many of them are based on qualitative modeling formalisms [29–31] that can deal with large networks, but are limited in their capacity to describe dynamic properties such as signal duration and amplitude. Interaction graphs as one example for qualitative models have been shown to be valuable tools for analyzing the structure of signaling pathways as they are implicitly contained as the underlying network structure in more complex modeling formalisms [32]. They can be used to make predictions on the possible qualitative behavior of a signaling network, and these predictions can be compared with experimental data. Resulting inconsistencies between data and network structure provide then a basis to identify missing or inactive interactions in the network structure. One possibility to derive such predictions is to use the concept of the dependency matrix [32, 33]. In contrast to related methods, which rely on the concept of sign consistency and require a steady state assumption [30, 34], exploitation of the dependency matrix is well-suited for the analysis of transient effects. The general idea is that some characteristics of the possible qualitative system response are determined by the paths and feedback loops that represent the interaction graph. Interaction graphs are closely linked to logical models. They can be derived from an interaction graph by adding rules that define how the discrete state of a node is governed by the states of other nodes [33]. While logical models are well-suited for studying the input-output behavior of large signaling pathways, an appropriate description of crosstalk mechanisms within the logical formalism is often difficult. This is due to the fact that, in contrast to the main activation routes of a signaling pathway, crosstalk mechanisms typically enhance or downgrade certain effects, rather than being necessary for or completely blocking them. Therefore, interaction graphs that utilize continuous states are preferable to describe crosstalk mechanisms.

To analyze the impact of crosstalk and feedback regulation, dynamic modeling approaches using coupled ordinary differential equations (ODEs) are most suited and allow quantitative insights [24, 35–38]. However, consideration and systematic analysis of a large number of potential mechanisms results in a high combinatorial complexity with many degrees of freedom and is therefore often not feasible with ODEs. Therefore, it is desirable to exploit the advantages of both qualitative and quantitative modeling and to develop strategies to combine both approaches.

**Fig 1:**
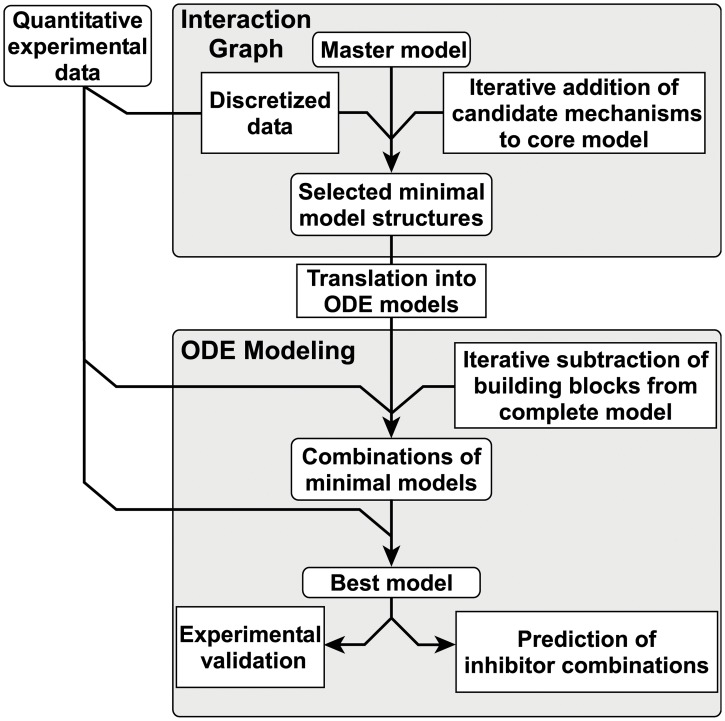
Workflow of model selection strategy. Quantitative time resolved data is discretized for the selection of submodels of the interaction graph master model. The interaction graph master model is based on literature knowledge and consists of the “core model” and reported interactions between the signaling pathways of interest, the “candidate mechanisms”. Ordinary differential equation (ODE) modeling utilizes the entire information of the time resolved data. Model selection based on parameter estimation permits the selection of the best model structure.

Here, we present a novel hybrid approach (Fig 1), which combines qualitative and quantitative modeling techniques to unravel the HGF induced activation of MAPK and PI3K signaling in primary mouse hepatocytes based on time-resolved experimental data. We started with an interaction graph master model containing previously reported crosstalk, feedback and feedforward mechanisms and selected then minimal model structures of the interaction graph master model that can explain the observed qualitative characteristics of the experimental data. In this way, the search space of model candidates was vastly reduced. With the subsequent analysis using ODE models, we identified the model structure representing the experimental system best. We demonstrate that the inferred HGF model shows robust behavior against single perturbations, but enables predictions of combinatorial inhibition leading to strongly reduced Akt and ERK activation. This application demonstrates the potential of our network inference approach to guide the development of context-specific therapeutic intervention strategies.

## Results

### HGF induced signaling pathways

**Fig 2:**
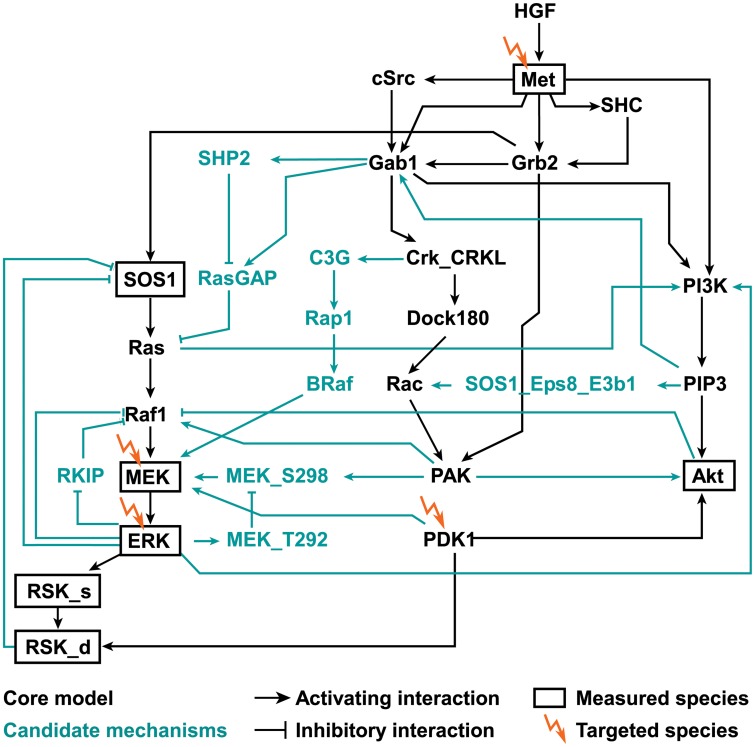
Interaction graph master model. The interaction graph master model was built from literature information. Detailed model documentation can be found in S1–S3 Tables. The core model is given in black, candidate mechanisms are depicted in turquoise. Arrows represent activating (positive) interactions, blunt-ended lines indicate inhibitory (negative) interactions. The measured species are marked with bold borders. The lightning symbol indicates that the respective species was experimentally targeted with a chemical inhibitor or siRNA.

To unravel the crosstalk between the MAPK and PI3K signaling pathways upon HGF stimulation in hepatocytes, we built an interaction graph model of HGF induced activation of these pathways based on literature knowledge (Fig 2). We distinguish between interactions that form the main activation routes (“core model”; black edges in Fig 2) and interactions describing feedback and crosstalk mechanisms (“candidate mechanisms”; turquoise edges) that have been reported for special cell types or under certain experimental conditions (S1–S3 Tables). The full graph including the core model and all candidate mechanisms is considered as a non-cell-type specific “HGF master model”.

**Fig 3:**
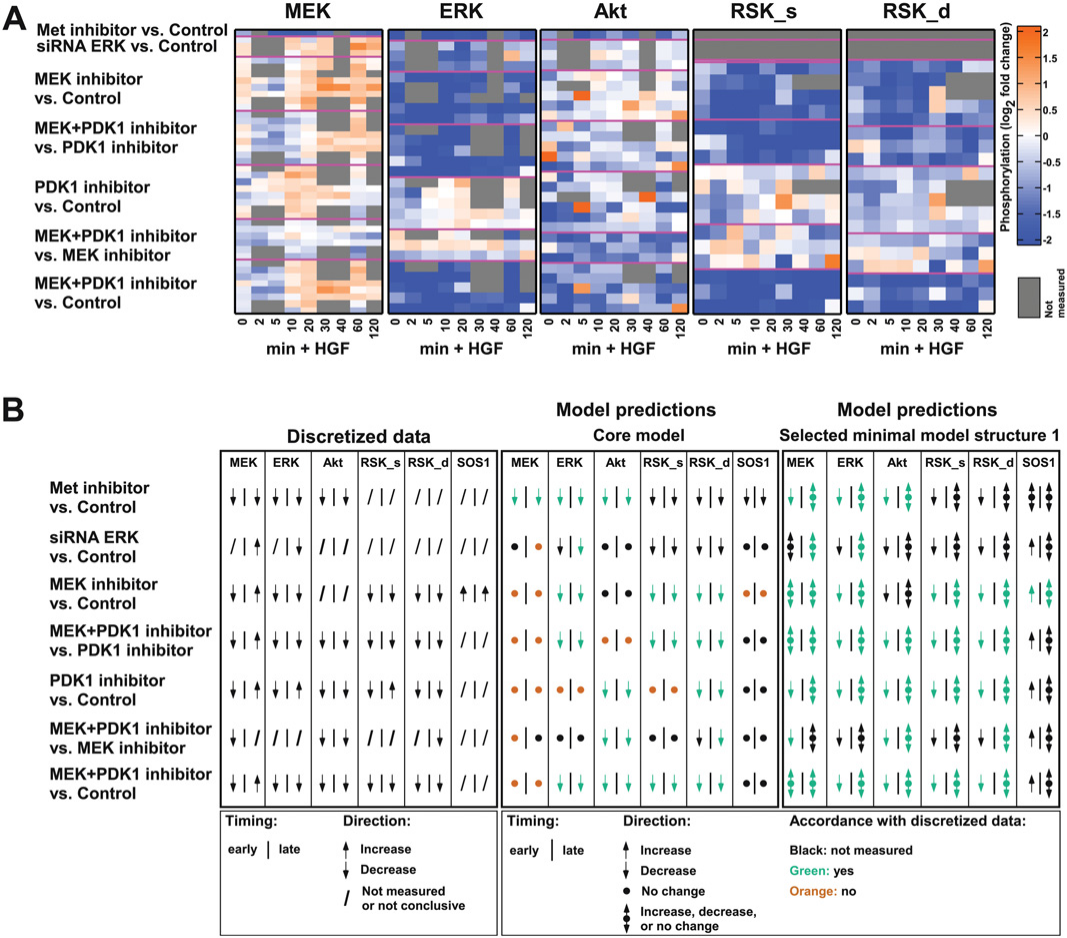
Experimental results and predictions by interaction graph models. A) For each indicated protein, the fold change of the phosphorylation state measured by quantitative immunoblotting of two different experimental conditions is shown on a logarithmic scale at the indicated time points after HGF stimulation. Each row refers to one experiment; same experimental conditions are grouped with magenta lines. B) The discretization (left panel) is based on Fig 3A and additional SOS1 measurements (S1A Fig). A slash shows that no measurements were taken or that the response was not conclusive. In the middle and right panel, predictions from the core model and, exemplary, from the identified substructure 1 (Fig 4) are shown. Arrow pointing up/down: the inhibition can only cause an increased/decreased activation of the measured protein. Bullet point: the inhibition does not affect the measured protein. Combined up/down arrow and bullet point: the model does not restrict the response to the inhibition.

To analyze which of the candidate mechanisms are active in our cellular system, we treated primary mouse hepatocytes with HGF in combination with specific Met inhibitor (PHA 665752), MEK inhibitor (U0126), PI3K inhibitors (LY294002, Wortmannin and PI-103) and PDK1 inhibitor (BX-912) alone or in combination. Additionally, we employed siRNA targeting Akt and ERK1/2. By time-resolved quantitative immunoblotting and by protein array we analyzed the phosphorylation of Akt, MEK1/2, ERK1/2 and p90RSK (S1 and S2 Fig). p90RSK activation occurs in two-steps: RSK_s denotes p90RSK phosphorylated at a single residue, RSK_d refers to the double phosphorylated, fully active form of p90RSK (S1 and S2 Tables). We also measured the dynamics of SOS1 activation upon MEK inhibitor treatment (S1A Fig). The fold change of protein phosphorylation for each treatment condition was calculated in comparison to the respective control and between treatment conditions (Fig 3A). For SOS1 activation, the band shift was quantified as an indicator for its activation status (S1A and S3A Figs). As expected, upon Met inhibitor treatment a strong decrease in phosphorylation of all measured proteins was detected. Upon inhibition of MEK, MEK phosphorylation was initially decreased, followed by an increased and sustained signal, whereas ERK, RSK_s and RSK_d phosphorylation was strongly reduced. MEK inhibitor also influenced the dynamics of SOS1 activation resulting in a more sustained activation (S1A and S3A Figs). During the entire observation period the effect of MEK inhibitor treatment on Akt phosphorylation was highly variable between the experiments. The siRNA targeting ERK1/2 showed a comparable effect on ERK and Akt as the MEK inhibitor. Treatment with PDK1 inhibitor reduced Akt phosphorylation on serine 473 and threonine 308 (Fig 3A and S3A Fig). Interestingly, a decreased phosphorylation of MEK, ERK, RSK_s and RSK_d upon PDK1 inhibitor treatment was observed at early time points and a subsequent increase at later time points. When applying MEK and PDK1 inhibitors simultaneously, we observed decreased phosphorylation of all measured proteins, but MEK phosphorylation showed an increased signal at later time points. A comparable phosphorylation response was elicited by combinatorial treatment with MEK and PDK1 inhibitors or with PDK1 inhibitor alone except for Akt phosphorylation, which was additionally decreased upon the combinatorial inhibitor treatment. The comparison between the combinatorial treatment with MEK and PDK1 inhibitor and MEK inhibitor treatment alone showed decreased phosphorylation on Akt, MEK and RSK_d. Treatment with PI3K inhibitor and with siRNA targeting Akt were not considered in our study due to the high variability observed in our results (S3B Fig).

### Selection of minimal model structures

To relate the data to the interaction graph model (Fig 2), we discretized the measured responses to “increase”, “decrease” and “not measured or not conclusive” (Fig 3B): If a certain effect was consistently observed for the replicates within the same time points, it was included as an observed effect in the scheme of the discretized data. Otherwise, the effect was considered “not conclusive” and not taken into account. The fourth possible value "no change" was not observed in our data set. Additionally, we included the timing of the response in the discretized data, classifying an observed effect as an early or late response. "Early" refers to the initial qualitative response of a node within 30 minutes after HGF stimulation. A response was termed “late” if the qualitative behavior at any successive time point is different from the initial (early) response (Materials and Methods). Thus, the late response characterizes the first effect that is opposite to the early response, or indicates that the qualitative response is similar for all time points.

Given an interaction graph model, we can predict the possible qualitative responses of the considered proteins for the given experimental conditions using the concept of the dependency matrix [33]. If the model predictions are in accordance with the discretized data, the given structure is able to reflect the experimental results. Fig 3B shows the possible qualitative responses predicted from the interaction graph core model. Comparing model predictions with the discretized data, the majority of observed behaviors were not represented by the core model. Hence, we conclude that some of the candidate mechanisms must be active in primary mouse hepatocytes. To identify minimal substructures of the HGF master model consistent with the data, we added single candidate mechanisms and combinations thereof to the core model and tested the resulting model structures for their ability to represent the observed effects. In order to select all minimal submodels of the interaction graph master model that can explain the observed effects from the experimental data (Fig 3B, left panel) and that contain the core model, we proceeded as follows: starting from the core model, we added one candidate mechanism at a time (that is, all edges making up the mechanism, S3 Table) and derived the model predictions for the resulting interaction graph structure as explained above. If all experimental observations were in accordance with these predictions, the structure was added to the list of selected minimal model structures. If not, we combined the respective structure with other candidate mechanisms whose sole addition to the core model was not able to explain the data. Again, we checked the ability of each resulting model structure to explain the data and either considered the structure as selected minimal model or added further candidate mechanisms. We ensured that only minimal model structures were selected, that is, no submodel of a selected model can explain the data. Furthermore, the procedure assures that all minimal model structures that are able to explain the data were found.

Thereby, all possible minimal model structures were selected, each being composed of the core model and a subset of the candidate mechanisms. Thus, among the possible candidate edges that may be relevant for the other cell types and conditions, we selected candidate mechanisms that are specific for the HGF induced MAPK and PI3K signaling pathways in primary mouse hepatocyte. The candidate edges that have not been selected by our approach might play a role in other cell types and for other conditions.

In total, we identified 16 minimal model structures that can equally well explain our experimental data (Fig 3B, right panel and S4 Fig). Notably, in some cases, the qualitative response is not restricted by the model structure: If a path between the inhibited and measured species includes a negative feedback loop, or if paths of both signs exist between the two nodes, the actual response depends on the strength of the different mechanism and, thus, cannot be predicted by a purely qualitative model.

**Fig 4:**
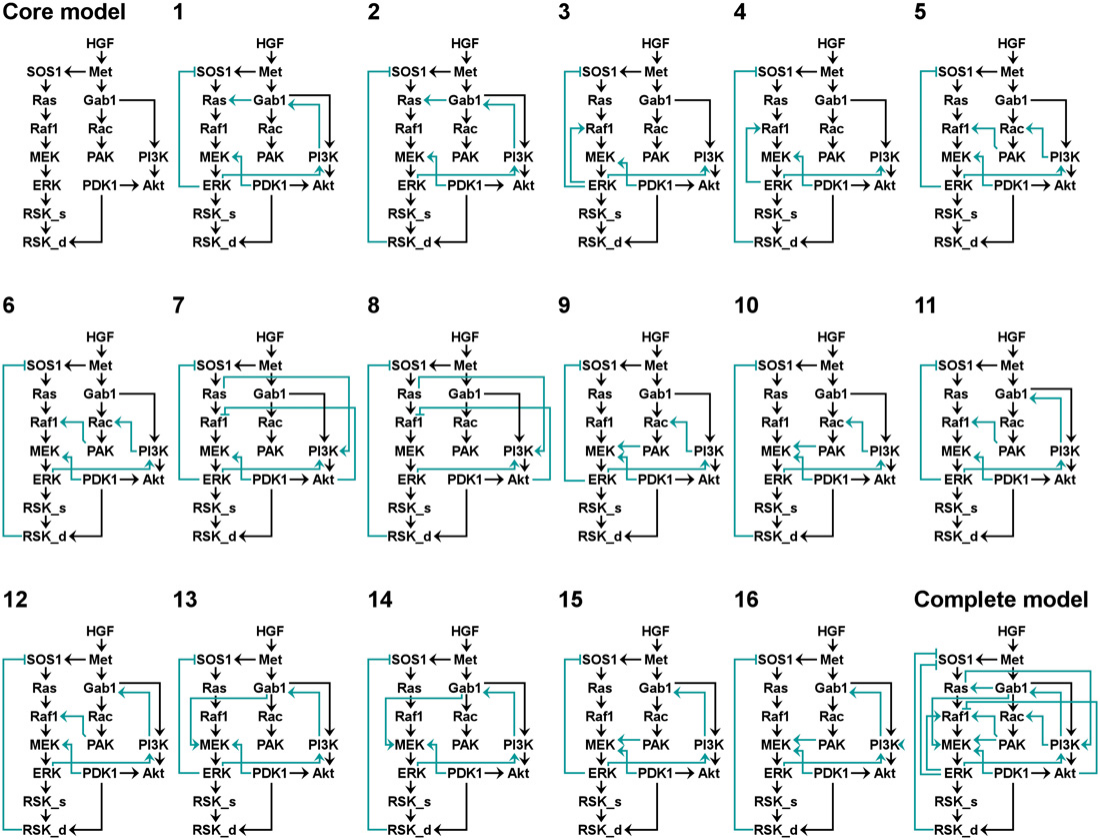
Selected minimal model structures, core and complete model. The compressed selected 16 minimal model structures that can explain the discretized data (Fig 3B) are shown. In addition, the complete model structure (that is the union of models 1–16) and the compressed core model structure are displayed. Arrows represent activating (positive) interactions; blunt-ended lines indicate inhibitory (negative) interactions. In each model, the core model is colored black, while the building block (the set of added candidate mechanisms) is shown in turquoise.

To develop corresponding ODE models with an appropriate number of parameters, we compressed all selected minimal model structures by removing parallel mechanisms with the same sign and by compressing linear chains [30]. Each candidate mechanism is represented by a single edge. Hence, each selected minimal model structure is composed of the compressed core model and a combination of candidate edges, each corresponding to one candidate mechanism. We define as “building block” the characteristic combination of candidate edges of a selected minimal model structure. Fig 4 shows the compressed selected 16 minimal model structures, the compressed core model and a model containing all building blocks (“complete model”). In contrast to the master model, the complete model contains only those candidate mechanisms that are included in at least one selected minimal model structure. By comparing the 16 minimal model structures, we observed that all candidate models include (i) the edge from ERK to PI3K, (ii) a negative feedback from ERK to SOS1, either directly or indirectly via RSK_d, (iii) a positive route from ERK to MEK through various mechanisms and (iv) a positive route from PDK1 to MEK. The latter route is included as a direct edge from PDK1 to MEK in all models but model 8, whereas model 8 contains a longer path via RSK_d, Ras and Akt.

### Ordinary differential equation model selection

**Fig 5:**
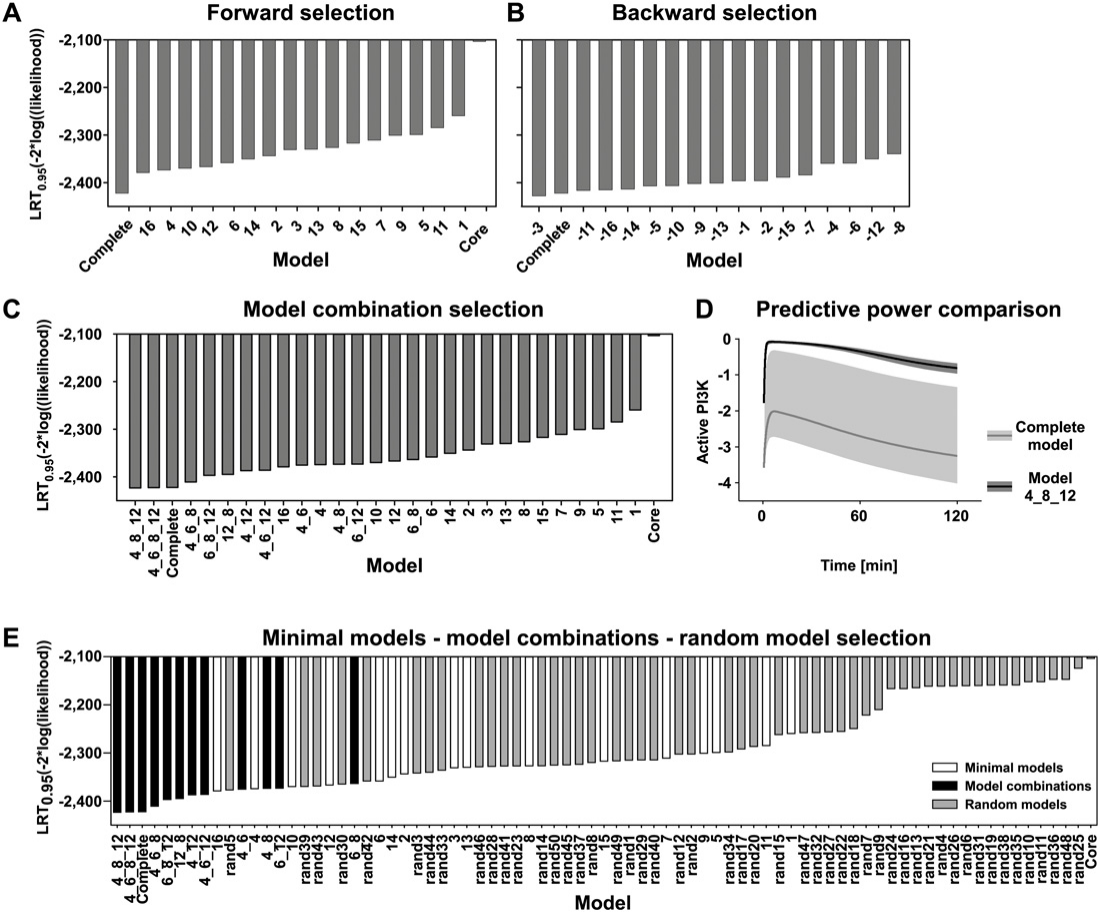
Model selection. A) Rankings represent the forward selection approach using selected minimal model structures; B) backward selection where the building blocks are removed from the complete model; C) the model combination selection. D) Comparison of the predictive power of the complete model and 4_8_12 model in respect of the kinetic of “active PI3K”. Confidence intervals of the predictions are indicated by shaded areas. E) Ranking of model selection including minimal model structures, model combinations and random models is shown. All rankings of model selection present the negative logarithmic likelihood penalized by parameter difference as described in Materials and Methods on the y-axis. Model identifiers are shown on the x-axis.

**Fig 6:**
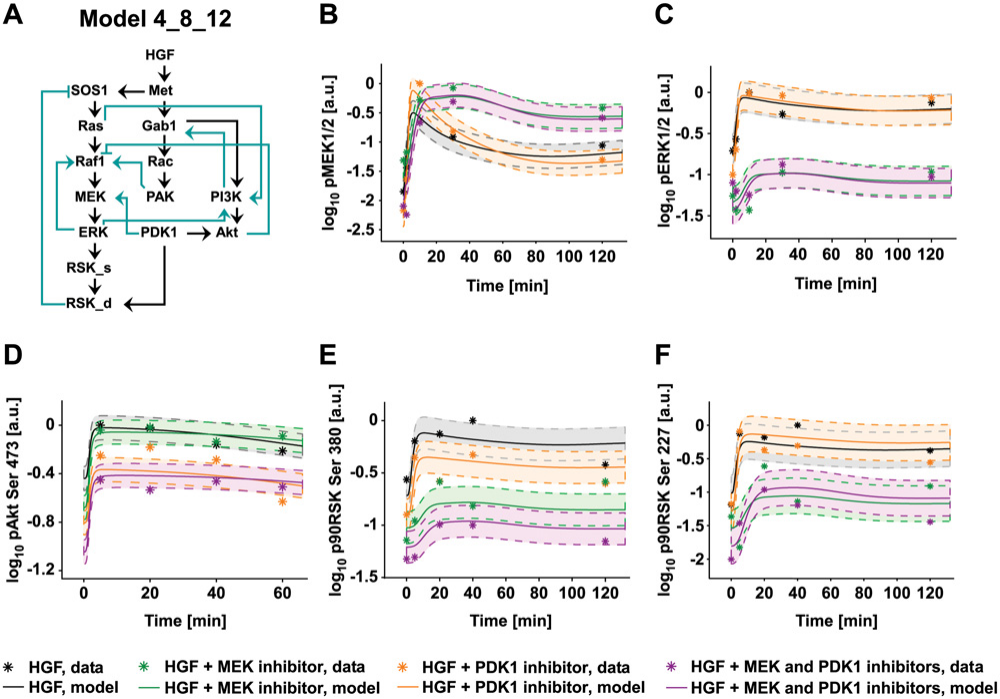
ODE model fit. A) Structure of the best performing model 4_8_12. B-F) Plots showing representative model trajectories (solid lines) of the phosphorylation kinetic of the indicated proteins measured by quantitative immunoblotting in primary mouse hepatocytes pretreated with the indicated inhibitors and stimulated with 40 ng/ml of HGF for the indicated time (stars). y-axes show the concentration of the respective measured protein in arbitrary units on a logarithmic scale. The shadowed area surrounding the model trajectory represents the confidence interval delimited by the dashed line. Treatments are color-coded as indicated in the figure.

To test which of the identified structures can quantitatively represent the transient and sustained effects observed in the experimental data, we translated each of the 16 compressed selected minimal model structures as well as the core and the complete model into an ODE model. While interaction graph modeling is based on discretized data, we utilized the full quantitative information of the data sets for ODE modeling (S1–S4 Datasets). In addition to the datasets used for the selection of the 16 model structures, we used datasets of time resolved measurements of HGF induced activation of the above mentioned proteins without inhibitor treatment. Furthermore, Met phosphorylation and active Ras measured by quantitative immunoblotting and protein array, respectively, were considered. In addition, the degree of phosphorylation of ERK and Akt after HGF stimulation was measured by mass spectrometry and included in the model. Due to its merely qualitative nature, the dataset on SOS1 activation was not used for ODE modeling. Overall, the ODE models were calibrated on 2200 data points and 25 experimental conditions including four targeted perturbations. A complete list of kinetic reactions considered for our modeling approach is shown in S4 Table. For each model structure, parameter estimation was performed to determine the model performance in relation to the experimental data. We applied an adaptation of a likelihood ratio test (LRT) with a threshold of 95%, which takes into account the different degrees of freedom for each model structure (Materials and Methods). This “forward selection” facilitated an LRT-based ranking of model structures (Fig 5A). As expected, the complete model structure performed best, whereas the core model structure performed significantly worse than all selected minimal model structures. Interestingly, the model ranking showed several selected minimal models with similar likelihood (16, 4 and 10). Therefore, a clear distinction of a best performing model structure was not possible. Furthermore, a significant gap was observed in the likelihood values between the complete model and the best performing selected minimal model structure, model 16. Thus, none of the minimal models is a valid simplification of the complete model. This result suggested that models containing combinations of building blocks might perform better than minimal models. To reduce the complexity of combining all model structures, we applied a “backward selection” based on the removal of single building blocks from the complete model structure followed by parameter estimation to obtain a new ranking (Fig 5B). Strikingly, the forward and backward selections revealed very different results. As an example, model 16 is the best performing selected minimal model structure in the forward selection, whereas a reduction of model 16 in the backward selection does not lead to a significant loss in likelihood. The removal of the selected minimal model structures 4, 6, 8 and 12 showed a significant loss in performance according to the likelihood, suggesting the importance of their building blocks. Based on the backward selection result, we generated combinations of model structures focusing on these four minimal models and performed parameter estimation for all eleven possible combinations (Fig 5C). As expected, the new ranking showed that these model structures filled the gap between the complete model and the best performing minimal model 16. Interestingly, combinatorial model structures 4_8_12 and 4_6_8_12 displayed a similar performance as the complete model structure, indicating that they are valid simplifications of the complete model. Between these models, model 4_8_12 performed best as it contained fewer parameters than model 4_6_8_12. The list of estimated parameter values of model 4_8_12 is given in S5 Table. A comparable ranking of all models was obtained utilizing the Akaike Information Criterion (AIC) (S6 Fig). Based on the ranking, we proposed a model structure consisting only of a subset of feedback and crosstalk mechanisms of the complete model, namely eight mechanisms out of thirteen. As shown in Fig 6B–6F, model 4_8_12 can reproduce the dynamic behavior of the measured species under diverse experimental conditions. A comparison of the performance of the core model, the complete model and the model 4_8_12 is given in S8 Fig. We hypothesized that the advantage of a reduced model resides in an improved predictive power. To compare the predictive power of the complete model and the model 4_8_12, we analyzed with the model the dynamic behaviour of a protein that has not been measured experimentally. We selected “active PI3K” that has not been used in the parameter estimation approach. We calculated the prediction profiles of “active PI3K” as described in [39] for the entire observation time frame. The prediction profiles were translated into confidence intervals along the trajectory, giving a measure of the accuracy of the prediction of the PI3K dynamic for both models. As shown in Fig 5D, model 4_8_12 outperforms the accuracy of the complete model by having an approximately 10-fold smaller confidence interval. In addition, the model trajectories of active PI3K differ significantly between the complete model and model 4_8_12. This indicates that prediction of the complete model is not only uncertain, but also incorrect as the confidence interval of the complete model does not contain the well-defined trajectory of model 4_8_12. This discrepancy is due to the parameter non-identifiabilites in the over-parameterized complete model. These results show that the selected reduced model 4_8_12 has a better predictive power than the complete model.

To challenge our approach, we generated 50 model structures consisting of randomly selected combinations of candidate edges. For the random model structures, we performed parameter estimation and derived a ranking as in the forward selection step (Fig 5E). This showed that the majority of random models performed worse than our selected models. Random models that performed well had a similar structure as the best performing selected models (S6 Table and Fig 5E). As an example, random model structure 5, which possesses three edges that are contained in model 12 or model 16, performed similarly as the selected minimal model structure 16. The two additional edges are present in model structure 4 and 8, respectively. Therefore, random model structure 5 is similar to the best performing model structure 4_8_12.

At first glance, model 4_8_12 includes three feedback loops (Fig 6A). Within the MAPK pathway, a positive and a negative feedback emerge from the candidate mechanisms ERK to Raf1 and RSK_d to SOS1, respectively. Within the PI3K pathway, the edge PI3K to Gab1 closes a positive feedback loop. Furthermore, model 4_8_12 is characterized by the presence of several crosstalk mechanisms between PI3K and MAPK. Notably, these mechanisms give rise to two positive and two negative feedback loops, each containing species from both the PI3K and MAPK pathways. As shown in Fig 6B–6F, model 4_8_12 can reproduce the dynamic behavior of the measured species under diverse experimental conditions. A comparison of the performance of the core model, the complete model and the model 4_8_12 is given in S8 Fig.

### Negative crosstalk: experimental validation

**Fig 7:**
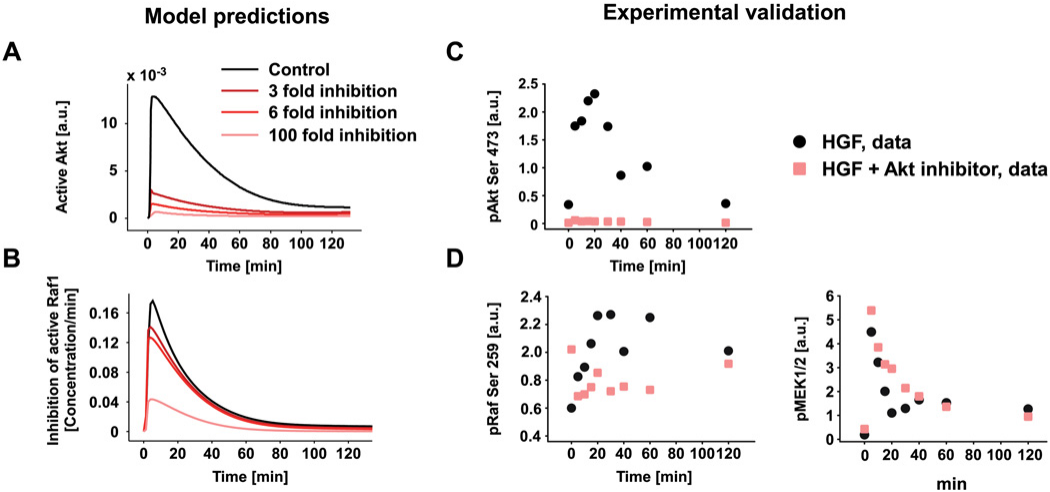
Negative crosstalk: experimental validation. A-B). Model prediction of active Akt and the loss of active Raf1 upon 3, 6 and 100 fold inhibition of active Akt. C-D) Experimental validation of the effect of Akt inhibition in primary mouse hepatocytes treated with 40 ng/ml of HGF alone or in combination with Akt inhibitor VIII. Quantification of the phosphorylation kinetics of Akt and Raf1 determined by quantitative immunoblotting (S9–S11 Fig).

To experimentally validate model 4_8_12, we focused on the identified interaction from Akt to Raf1 and predicted the impact of different degrees of Akt inhibition on the inhibitory impact of Akt on Raf1. The model predictions indicate that 3, 6 and 100 fold Akt inhibition results in a 77%, 83% and 99% reduction of the inhibitory effect of Akt on Raf1 (Fig 7A and 7B). The model includes active Raf1, which cannot be directly compared to Raf1 phosphorylated at a specific phosphorylation site as phosphorylation on serine 338 contributes to Raf1 activation, whereas phosphorylation on serine 259, the target of Akt, represents an inactivation signal [40]. Therefore, we used the effect of Akt inhibition on serine 259 phosphorylation of Raf1 as a proxy to experimentally address the inhibitory impact of Akt on Raf1. Experimentally, we stimulated primary mouse hepatocytes with HGF in the absence and presence of the specific Akt inhibitor VIII and monitored the impact on Raf1 phosphorylation on serine 259. As shown in Fig 7C and S9–S11 Figs, we achieved an inhibition of Akt phosphorylation between 90% and 100% and concomitantly observed a decrease of Raf1 phosphorylation on serine 259 (Fig 7D and S9–S11 Figs). Additionally, a moderate increase of MEK phosphorylation is also observed upon Akt inhibitor treatment (Fig 7D). The results confirmed the presence of this interaction in our cellular model system upon HGF stimulation. This interaction is also present in models 7 and 8, but these models have not been selected as best performing models. We emphasize that, since model 4_8_12 is derived from the combination of the single models 4, 8 and 12, it does not contain unique edges. However, the validation of model 4_8_12 as the optimal network structure in primary mouse hepatocytes is given by our model selection process together with the experimental validation of the interaction between Akt and Raf1.

The candidate mechanisms within model 4_8_12 generate crosstalk as well as feedforward and feedback loops within the network structure leading to robust network behavior. These characteristics may limit the efficacy of targeted therapies or create undesired effects. Therefore, to further validate our model structure, we analyzed how the system responds to different perturbation conditions.

### Inhibitor combination: model predictions and experimental validation

**Fig 8:**
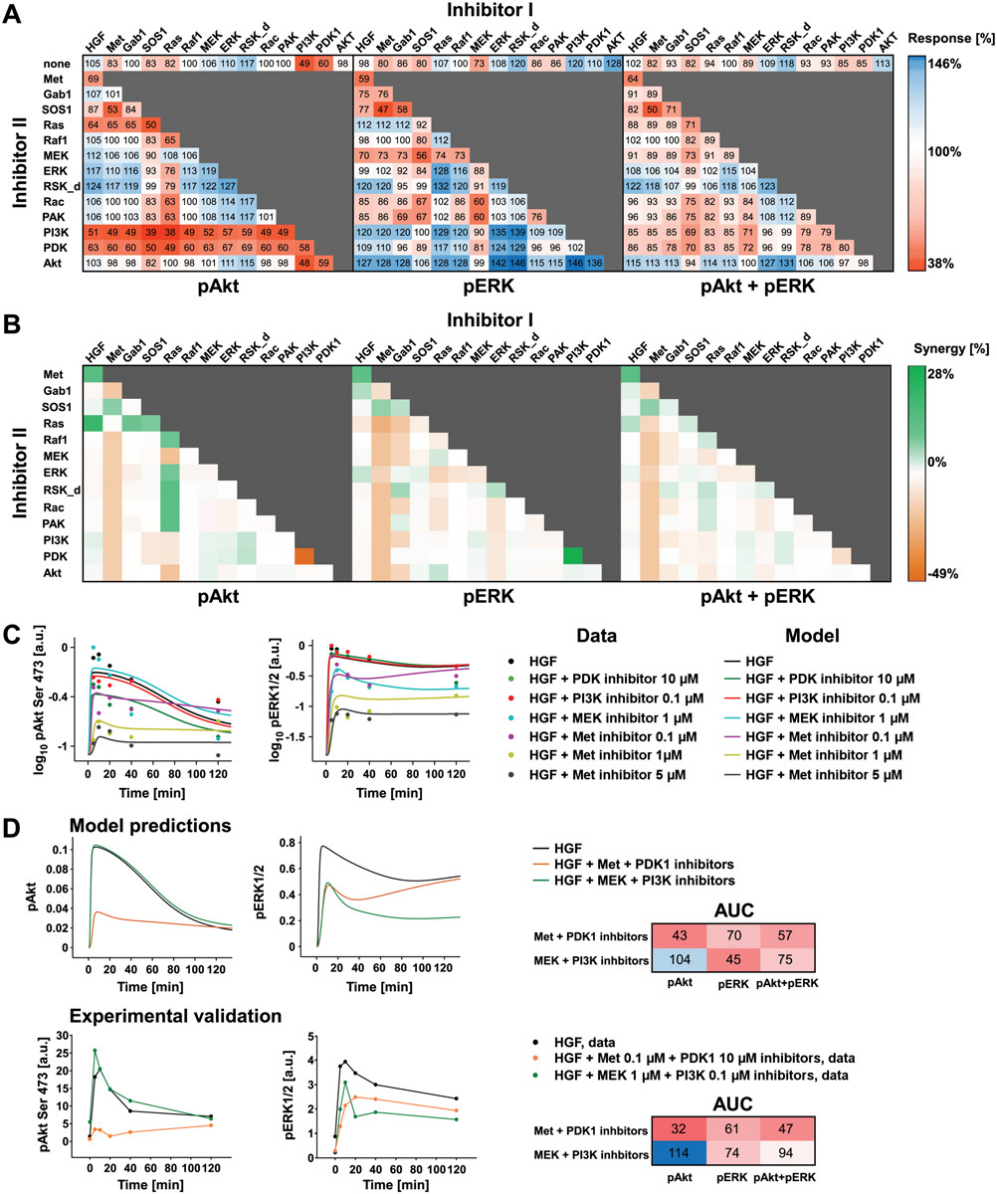
Inhibitor combination: model predictions and experimental validation. A) Heatmaps showing model simulations of the impact of 50% inhibitor I individually or in combination with 50% inhibitor II. As readout, the area under the curve of pAkt, pERK, and the sum of pAkt and pERK upon inhibitor treatment is compared to the area under the curve of the control condition. The change in the response induced by the inhibitor treatment is indicated as percentage to the control condition. B) Heatmap of synergistic effect of inhibitor combination treatment shown in panel (A). The synergy represents the efficiency of the double inhibitor treatment compared to individual inhibitor treatments. C) Inhibitor strength parameter estimation. Model 4_8_12 trajectories (solid lines) of the phosphorylation kinetic of pAkt and pERK measured in primary mouse hepatocytes treated with the indicated inhibitor or DMSO prior to HGF 40 ng/ml treatment (filled circles). The experimental data represent the average of two or more replica. D) Model predictions of pAkt and pERK kinetics and experimental validation of inhibitors combination treatment. Model predictions are based on the inhibitor strength estimated as in Fig 5C. The experimental validation is based on primary mouse hepatocytes treated with the indicated inhibitors or DMSO and subsequently stimulated with 40 ng/ml HGF for the indicated time points. Quantification of the phosphorylation kinetics of Akt and ERK determined by quantitative immunoblotting (S12 Fig). Quantification of the area under the curve (AUC) of pAkt, pERK and their sum is indicated for the model trajectories and the experimental data. The experimental data is a representative dataset of an experiment performed in biological duplicates.

To identify strategies to efficiently inhibit Akt and ERK signaling, we used the inferred 4_8_12 model to determine the effect of reducing the rate of outgoing reactions of each protein by 50%. Additionally, we performed simulations for every possible combination of double inhibition. As readout of PI3K and MAPK pathway activation, we calculated the area under the curve of Akt and ERK phosphorylation and the sum thereof to evaluate the effect of the targeted inhibition (Fig 8A). Single inhibition of PI3K or PDK1 drastically reduced pAkt and, surprisingly, exerted the opposite effect on pERK. This result indicated that the network structure was robust against single inhibitor treatment suggesting that the two signaling pathways compensated each other. We calculated the sum of the integrals of Akt and ERK phosphorylation to predict the double inhibition resulting in an effective reduction of both readouts (Fig 8A). Interestingly, while the sum of the integral of pAkt and pERK is sensitive to the combined inhibition of PI3K and MEK resulting in a 71% reduction of the sum, the combined inhibition of Met and RSK results in an increase of the sum to 118%. In some cases, we observed synergistic effects as the combination of inhibitors had a stronger effect on the readout than the sum of impact of the respective individual inhibitions. For example, single inhibition of Met or SOS1 led to the reduction of the sum of the integral of pAkt and pERK by 18%, while their combination led to 50% reduction. To address the synergistic effect of the inhibitor combination treatment, we calculated the effect of the inhibitor combination compared to the single inhibitor treatment (Fig 8B). Interestingly, we observed that the Ras inhibitor has a synergistic effect on pAkt and pERK with several other inhibitors such as Raf1, ERK and Rac inhibitors. We furthermore detected synergy between the PDK1 and Akt inhibitor on pAkt and between the Met inhibitor and the SOS1 inhibitor on pAkt and pERK. Surprisingly, the combination of PI3K and PDK1 inhibitors shows the lowest synergistic effect on pAkt and the highest on pERK, resulting in a mild synergy in the sum of pAkt and pERK.

To experimentally validate the model predictions on combinatorial treatments, we first performed single inhibitor treatments targeting PDK1, PI3K, Met and MEK prior to HGF stimulation and estimated the inhibition strength parameters for each individual inhibitor for model 4_8_12 (Fig 8C). We estimated 10% strength for the PI3K inhibitor, 83% for the MEK inhibitor, 90% strength for the Met inhibitor, and 53% for the PDK inhibitor. Based on the estimated inhibitor parameters, we performed predictions of the dynamic behavior of pAkt and pERK upon combinatorial inhibitor treatments. In detail, we simulated the activation kinetics of pAkt and pERK upon combining the PI3K and the MEK inhibitor and upon combining the Met and the PDK1 inhibitor. We experimentally validated the model predictions by treating primary mouse hepatocytes with the indicated inhibitor doses and combinations and analyzed the activation kinetic of pAkt and pERK (Fig 8D and S12 Fig). The experimental results indicate that the combination of low dose of the PI3K and the MEK inhibitor slightly increases pAkt and reduces pERK and, therefore, are in good agreement with the model predictions. Interestingly, the combined application of the Met and the PDK1 inhibitor resulted in a reduction of pAkt, while it had only a moderate effect on pERK. Additionally, the calculated area under the curve of pAkt, pERK and their sum for the model trajectories and the experimental data are in agreement. In conclusion, model simulations and experimental verifications suggested that the considered signaling network is less sensitive to single interventions, but can be efficiently targeted by combinatorial treatments.

## Discussion

The identification and quantitative description of relevant feedback, feedforward and crosstalk regulation of signaling pathways is an important step towards understanding cellular signaling networks and a key prerequisite for the development of successful drug targeting strategies [41–43].

Our network inference approach combines qualitative (interaction graph) and quantitative (ODE) modeling and, hence, benefits from the strengths of both methods. The key advantage of our introduced hybrid approach is that interaction graph modeling enables pre-selection of minimal model structures from a vast search space of potential model candidates, which are then translated into ODE models for integration of quantitative details. Several other mathematical modeling approaches also deal with a family of candidate models aiming at the identification of the correct wiring. These approaches employ, for example, ensemble modeling [37] or Bayesian inference [44] and can usually only deal with a limited number (tens to hundreds) of competing ODE models. However, if one considers all possible combinations of reported crosstalk, feedback and feedforward mechanisms, these possibilities result in an enormous number of candidate models. As shown herein, by pre-selecting minimal model structures using interaction graph modeling, one can massively reduce the search space (here from 10^5^ to 16) before continuing with a smaller library of ODE models. Other modeling approaches using perturbation data to unravel the network structure rely on modular response analysis (MRA), which requires steady state assumptions and linear equation based modeling. Therefore, these approaches are more suited to identify feedback and crosstalk mechanisms that determine the medium to long term behavior [24, 35]. Furthermore, a complete set of perturbation experiments that alter the state of each individual module is required. In contrast, our proposed modeling strategy also enables the elucidation of mechanisms influencing the immediate system response as well as taking into account non-linear effects such as saturation.

Applying our approach to HGF signaling in primary mouse hepatocytes, the ranking of the resulting minimal ODE model structures by forward selection indicated that (i) model structures harboring the negative feedback edge from RSK_d to SOS1 in most cases outperformed their partner model containing the negative feedback edge from ERK to SOS1 and (ii) random model structures, which do not include any of these negative feedback edges, are strongly outperformed by the candidate models. The presence of a negative feedback within the MAPK signaling is well known, and ERK is usually considered as central player of this feedback [45]. However, in line with our findings, it was shown that the negative feedback to SOS1 could also be mediated by p90RSK [4]. Although forward selection did not allow us to identify a single selected minimal model structure as the one explaining the experimental data best, these structures served as important basis for further analysis. By backward selection, we identified minimal model structures containing feedback and crosstalk mechanisms relevant for HGF mediated responses in primary mouse hepatocytes resulting in a reasonable number of combinations of minimal model structures that had to be tested. The best performing combinatorial model 4_8_12 showed a positive feedback edge of ERK to Raf1. This feedback is known to involve RKIP [5], and it is the result of two negative effects: RKIP negatively regulates MEK activation by binding to phosphorylated Raf1 and, therefore, inhibiting the signaling cascade activation. A negative feedback loop on RKIP is triggered by ERK allowing the release of Raf1 from RKIP-Raf1 complex resulting in MEK activation. This represents an additional mechanism to fine-tune signaling responses within the negative feedback from downstream proteins to SOS1. Furthermore, our approach unraveled a negative interaction from Akt to Raf1, which we confirmed experimentally in our cellular model system. Raf1 phosphorylation on serine 259 by Akt has also been shown in the HGF stimulated human hepatoma cell line Hep3B [46]. Raf1 phosphorylation on serine 259 contributes to control the mitogenic response induced by MAPK signaling [47], and it has been observed in the context of genetic disorders [48]. Finally, a crosstalk mechanism from ERK to PI3K is indicated in the model 4_8_12, which gives rise to two additional feedback loops controlling both the MAPK and the PI3K branch. The negative feedback loop involves the negative effect of Akt on Raf1; the positive feedback loop is mediated by PI3K activated Gab1 that strengths the MAPK activation via Rac and PAK.

Thus, our proposed network structure shows positive and negative feedback loops that are highly interlinked. Feedback loops are the base of non-trivial dynamic behavior. Positive feedback loops have been shown to cause bistable behavior [49] and must be contained in the system's structure to enable more than one steady state [50]. Negative feedback loops are known to stabilize the system's response [51] and are a structural prerequisite for oscillations [52]. Within the last decades, it has become more and more evident that complex and robust dynamics of cellular signaling networks arise from the interplay of positive and negative feedback loops [53]. Examples include that of periodic calcium spikes after growth factor or hormone stimulation [54] and the MAPK system, where positive and negative feedback loops allow the system to switch between monostable and bistable regimes and, therefore, to flexibly adapt the response to different stimuli [55].

Components of the MAPK and PI3K signaling pathways are frequently altered in pathological conditions and, thus, there is much interest in developing targeted therapies [56, 57]. A number of potential interactions between these two pathways have been studied in different cell types (S3 Table). By employing mathematical modeling, a study of the MAPK and PI3K pathways crosstalk showed that both compensate for each other [28]. Although these two pathways are well studied, a systematic strategy to unravel their crosstalk has been missing.

Our modeling strategy delivers as output a dynamic ODE model validated with respect to both parameters and network structure, as an invaluable tool to not only elucidate cellular signaling pathways, but also to design cell-type or even patient-specific intervention strategies. In our case study, various interconnections between the PI3K and MAPK pathway in the best performing model structure indicate redundancy within the HGF stimulated signaling network. Our model predictions suggest that the effects of intervention in one signaling pathway can be compensated by the impact of the other pathway, as in case of treatment with single PI3K or PDK1 inhibitor. To maximize the response of inhibition, combinatorial treatments are required as suggested previously [24, 36]. However, a positive synergistic effect is predicted only for some combinatorial treatments suggesting robustness. Therefore, analyses focusing on the identification of the most promising synergetic effects are essential to develop novel combinatorial treatments.

Our proposed strategy to disentangle complex signaling networks provides a generic and systematic approach to elucidate cell- and context-specific feedback and crosstalk mechanisms and promises to significantly improve the development of effective combinatorial intervention strategies.

## Materials and Methods

### Ethics statement

All animal experiments were approved by the governmental review committee on animal care of the state Baden Württemberg, Germany (reference number A24/10). Anesthesia was carried out by intraperitoneal injection of 5 mg ketamine hydrochloride 10% (w/v) (Bayer Health Care, Leverkusen, Germany) per 100 mg body weight and 1 mg xylazine hydrochloride 2% (w/v) (Pfizer, Berlin, Germany) per 100 mg body weight.

### Isolation of primary mouse hepatocytes

Mice of 8–12 week-old C57BL/6N (Charles River, Sulzfeld, Germany) were used for primary hepatocyte isolation. Mice, which were housed at the DKFZ animal facility under a constant light/dark cycle, maintained on a standard mouse diet and allowed *ad libitum* access to food and water, were used for primary hepatocyte isolation. Primary mouse hepatocytes were isolated as described [58]. After isolation, hepatocytes were seeded in full medium (phenol red-free Williams E medium (Biochrom) supplemented with 10% (v/v) fetal bovine serum (Life Technologies), 0.1 μM dexamethasone, 10 μg/ml insulin, 2 mM L-glutamine (Life Technologies) and 1% (v/v) penicillin/streptomycin 100x (Life Technologies)) using collagen I-coated cell dishes (BD Biosciences). Hepatocytes were cultured at 37°C, 5% CO_2_ and 95% rH. Following cell adhesion, hepatocytes were washed with PBS (PAN Biotech) and subsequently cultivated in serum-free cultivation medium (phenol red-free Williams E medium supplemented with 0.1 microM dexamethasone, 2 mM L-glutamine and 1% (v/v) penicillin/streptomycin 100x) for 14 hours. Subsequently, hepatocytes were washed with PBS (PAN Biotech) and cultivated in serum-free cultivation medium depleted of dexamethasone for 6 hours prior treatment.

### Time course and dose response experiments

For time course and dose response experiments after isolation, hepatocytes were seeded at confluence (2x10^6^ cells/6 cm dish) and cultivated as described before. For mass spectrometry analysis, after isolation, hepatocytes were seeded at confluence (5x10^6^ cells/10cm dish) and treated as described [59]. After 6 hours in serum-free cultivation medium depleted of dexamethasone, hepatocytes were stimulated with indicated concentration of recombinant mouse HGF (HGF) (R&D Systems) at indicated time points (Supporting Information Datasets). For inhibitor experiments, cells were treated for 30 to 45 minutes with 20 microM MEK inhibitor U0126, 5–25 microM PI3K inhibitors LY294002, Wortmannin (all Cell Signaling Technologies), or PI-103 (Calbiochem), 20 microM PDK1 inhibitor BX-912 (Axon Medchem), 20 microM Akt inhibitor VIII (Millipore), or 5–20 microM Met inhibitor PHA-665752 (TOCRIS Bioscience) prior to stimulation with 40 ng/ml of HGF for the designated time points. The control condition was carried out by treating the cells with dimethyl sulfoxide (DMSO, Sigma-Aldrich). Model validation experiments were carried out by stimulating the primary mouse hepatocytes with the following inhibitor combinations: 1 microM MEK inhibitor U0126 (Cell Signaling Technologies) with 0.1 microM PI3K inhibitor PI-103 (Calbiochem); 5 microM MEK inhibitor U0126 (Cell Signaling Technologies) with 0.5 microM PI3K inhibitor PI-103 (Calbiochem); 10 microM PDK1 inhibitor BX-912 (Axon Medchem) with 5 microM Met inhibitor PHA-665752 (TOCRIS Bioscience); 10 microM PDK1 inhibitor BX-912 (Axon Medchem) with 1 microM Met inhibitor PHA-665752 (TOCRIS Bioscience); 10 microM PDK1 inhibitor BX-912 (Axon Medchem) with 0.1 microM Met inhibitor PHA-665752 (TOCRIS Bioscience). Single inhibitor treatments were also performed using the inhibitor concentrations listed before. All inhibitors were applied 30 minutes prior to stimulation with 40 ng/ml of HGF for the designated time points.

For siRNA treatment after isolation, hepatocytes were seeded at subconfluence (900000 cells/well in 6 wells/plate). After adhesion, cells were washed as described above and serum-free cultivation medium was added. Subsequently, hepatocytes were transfected with siRNAs by applying RNAiMAX (Invitrogen) and incubated for 16 hours. ON-TARGET*plus* SMART Pool siRNA targeting mouse Akt, ERK1 and ERK2 (Dharmacon, Thermo Scientific) were applied to a final concentration of 10nM, (ERK1 and ERK2 ON-TARGET*plus* SMART Pool siRNAs were pooled). The control condition was carried out by using ON-TARGET*plus* Non-targeting Pool. After 16 hours, cells were washed, and serum-free cultivation medium depleted of dexamethasone was added 6 hours prior stimulation.

### Quantitative immunoblotting

Cells were lysed on ice at the indicated time points using total cell lysis buffer (20 mM Tris pH7.4, 150 mM NaCl, 1mM EDTA pH8.0 (AppliChem), 1% (v/v) NP40 (Roche Applied Sciences), 1mM ZnCl_2_, 1mM MgCl_2_, 1 mM Na_3_VO_4_, 10 mM NaF, 10% glycerol, 0.1 mg/ml AEBSF, 1μg/ml aprotinin). Total cellular lysate was used to assay MEK, ERK, Akt, Raf1, or they were subjected to immunoprecipitation (IP) using 500 micrograms of total protein and subsequently assayed on SDS-PAGE. For IP anti-Met, aanti-Raf (both Santa Cruz Biotechnology) and anti-SOS (Merk Millipore) antibodies were used. Samples were loaded in a random order to reduce the correlated error [60]. Primary antibodies anti-p44/42 threonine 202/tyrosine 204 phosphorylation (anti-pERK1/2), anti-p44/42 total protein (anti-ERK), anti-MEK serine 217/221 phosphorylation, anti-MEK total protein, anti-Akt serine 473 phosphorylation, anti-Akt threonine 308 phosphorylation, anti-Akt total protein, anti-p90RSK serine 227 phosphorylation, anti-p90RSK 380 phosphorylation, anti-p90RSK total protein (Cell Signaling Technology), anti-Raf serine 259 phosphorylation (Cell Signaling Technology), anti-Raf serine 338 phosphorylation (Upstate), anti-Raf total protein (Santa Cruz Biotechnology), anti-SOS (Merk Millipore), anti-phosphotyrosine 4G10 (Merck Millipore), anti-PDI (Upstate), anti-actin (Sigma) were used. For the quantification of active Ras, cells were lysed with total cell lysis buffer (125 mM HEPES (pH 7.5), 750 mM NaCl, 1% NP40, 50 mM MgCl_2_, 10% glycerol, 0.1 mg/ml AEBSF, 1 microgram/ml aprotinin and protease inhibitor cocktail (Roche)). The whole lysate was subjected to IP supplemented with MgCl_2_ (50 mM) and Raf-RBD-GST-beads or processed on protein array (as described in the protein array section). After the IP, the lysates were assayed on SDS-PAGE and primary antibody anti-panRas (OP21) (Calbiochem) was used for detection. Secondary horseradish peroxidase-coupled antibodies (anti-rabbit HRP and anti-mouse HRP) were used (Amersham Biosciences). Target proteins were visualized using enhanced chemiluminescence (GE Healthcare), and signals were acquired using the CCD camera-based device LumiImager (Roche Diagnostic) or ImageQuant LAS 4000 biomolecular imager (GE healthcare). Immunoblot data were quantified using LumiAnalysis software (Roche Diagnostic) or ImageQuant TL version 7.0 software (GE Healthcare).

### Mass spectrometry

Primary mouse hepatocytes were isolated and cultivated as described above. For Akt and ERK phosphorylation analysis, after 6 hours of serum-free cultivation medium depleted of dexamethasone, the cells were stimulated with 40 ng/ml or 100 ng/ml of HGF for 10 minutes or left untreated. Total cellular lysate was denaturated in 10% SDS and subsequently subjected to IP of ERK1 and Akt (Santa Cruz Biotechnology). For IP, anti-ERK1 (Santa Cruz Biotechnology) and anti-Akt (Cell Signaling Technology) antibodies were used. Immunoprecipitated proteins were resolved on SDS-PAGE, and gels were stained with SimplyBlue SafeStain Life Technologies according to manufacturer’s instructions. Quantitative determination of the degree of phosphorylation of Akt and ERK was performed as previously described [59, 61] and was used for ODE model calibration.

### Protein array: Akt and ERK quantification

Protein array analysis was performed for the simultaneous detection of Akt and ERK phosphorylation. The spotting was performed with a sciFLEX-Arrayer SP5 (Scienion, Berlin) piezoelectric non-contact spotter on 16-pad nitrocellulose slides (Oncyte, Grace). For Akt detection, the following non-rabbit-derived capture antibodies were used: sc-55523 (Santa Cruz Biotechnology) and CS2967 (Cell Signaling Technology) for total Akt, Up05669 (Upstate) for phosphorylated Akt. For ERK detection, the following spotting antibodies were used: BD610030 (BD Bioscience), Up05157 (Upstate), MAB1576, CS9107 (Cell Signaling Technology) for total ERK and CS9106, CS9109 (Cell Signaling Technology), M9692, for phosphorylated ERK. Bovine serum albumin from Sigma (A9418) was labelled with the DyLight800 fluorophore using DyLight 800-NHS-Ester (46422) and Fluorescent Dye Removal columns (22858) from Thermo Scientific were used for the dilution of the capture antibodies for a spot to spot normalization. Capture antibodies (diluted the following: M9692 (1:8 dilution), BD, CST, Sigma, Upstate (1:3,6 dilution), Santa Cruz Biotechnology undiluted) were mixed with 2x Whatman arraying buffer (S00537), containing a 1:100 dilution of DyLight800-labelled BSA to account for spotting differences. Spotted slides were blocked with Odyssey blocking buffer (Licor 927–40000) for 6 hours shaking at 4°C.

Recombinant proteins were used as calibrators. Commercially available phosphorylated ERK (PV3313, Life Technologies); unphosphorylated ERK (PV3312, Life Technologies) and phosphorylated Akt (14–276, Millipore) and unphosphorylated Akt (14–279, Millipore) were diluted for different dilution series, supplemented with 1% SDS and denatured for 5 minutes at 95°C. Cell lysates generated as for quantitative immunoblotting were diluted 1:16 in Array Buffer Plus (Array Buffer composed by 1% BSA, 0,5% NP40, 0,02% SDS, 50mM Tris pH7,4, 150mM NaCl, 1mM EDTA, 5mM NaF, 1mM Na_3_VO_4_) containing complete protease-inhibitor cocktail (Roche), supplemented with SDS and denatured. Slides were incubated with calibrators and cell lysates shaking at 4°C overnight. After washes with the Assay Buffer at room temperature the slides were incubated with the detection antibodies for 2 hours shaking at 4°C. Detection antibodies were used for total ERK Up06-182 (Upstate), sc-94 (Santa Cruz Biotechnology) diluted 1:800 and total AKT sc-1619 (Santa Cruz Biotechnology) diluted 1:200, CS9272 (Cell Signaling Technology) diluted 1:400. After washes with Array Buffer and Wash Buffer (PBS (Pan, ready-made), NP-40 (0.2%), NaF (5 mM), Na-Vanadate (1 mM)) the slides were incubated with the secondary detection antibody (goat anti-rabbit coupled to Alexa Fluor 680) for 30 minutes shaking at 4°C. Slides were light-protected and washed with Wash Buffer and ddH_2_O and subsequently dried. Slides were scanned with LiCor Odyssey scanner at 700 and 800 nm (Resolution 21 μm, High Quality, Intensity: 5/4; Offset: 0, size 7x5 Scanning time approx. 35min/2slides). The quantification of the signal was performed as described [61].

### Protein array: Ras quantification

We generated protein array analysis to quantify active Ras by using a mouse antibody (Pan-Ras, OP40, Calbiochem) and GST-tagged Ras-binding-domain (RBD) of Raf (GST-Raf-RBD 1–149) [56] for Ras detection. To generate the pGex plasmid harboring the GST-tagged Raf-RBD we cloned the Raf-RBD into the pGex vector via BamHI and EcoRI sites. The pGex-GST-Raf-RBD was expressed in BL21 bacteria and the fusion protein was purified using Gluthatione Sepharose beads (GE Healthcare). To spot the eluted fraction, it was diluted with PBS and glycerol. The spotting was performed as described in the previous paragraph. Cell lysates were diluted 1:10 with Array Buffer PLUS. For calibration positive and negative control samples with gammaS-GTP and GDP loading were used. Prior to incubation, the slides were blocked with LiCor Blocking Buffer for 2–6 h. Samples and calibrator-solutions were incubated on the slides shaking overnight. All incubations were performed at 4°C. The slides were then washed with array buffer and incubated with specific rabbit-derived detection Ras antibody sc-14022 and sc-520 (Santa Cruz Biotechnology). Slides were subsequently treated as described in the previous paragraph.

### Interaction graphs

Interaction graphs are an abstract representation of signaling pathways [32]. The nodes in these graphs represent the signaling molecules, in our application usually the activated forms of the proteins (S1 Table). Directed positive and negative edges represent activating or inhibiting influences between the proteins.

#### Selection of minimal model structures from the interaction graph

We identified all minimal submodels from the interaction graph master model that can explain all qualitative properties of our experimental data based on the concept of the dependency matrix [33]. In the dependency matrix, the effect of one species on another is classified as follows: If all paths from species A to species B are positive, A is an activator of B. If all paths from A to B are negative, A is an inhibitor of B. If positive and negative paths lead from A to B, A is said to be an ambivalent factor for B, and A is a neutral factor for B if there are no paths from A to B. Activators and inhibitors are further classified as weak or strong depending on whether any of the paths runs through a node that is involved in a negative feedback or not [33]. Weak and strong activators/inhibitors differ in how they restrict the possible qualitative behavior. Decreasing the activity of a strong activator of species B leads to a decrease of the activation of B at every time point. In contrast, decreasing the activity of a weak activator of species B also leads to an initial decrease of the activity of B, but the negative feedback may cause a subsequent increase. This concept allows deriving predictions of the possible qualitative behavior in response to inhibitors given a certain model structure. To select the minimal model structures, we used the discretized fold change (‘increase’, ‘decrease’, ‘no change’, ‘not conclusive’) of the phosphorylation state of two experimental conditions, C1 and C2. C1 represents the “control condition”, whereas in condition C2, one or two additional inhibitors compared to C1 are applied (Fig 3B, left panel). For each comparison, we checked in the dependency matrix of the model how the additional inhibitors in C2 act on the measured species leading to qualitative predictions for the early and late differential behavior of this species in condition C2 versus C1. If all additionally inhibited species in C2 are activators of the measured protein, no matter whether they are weak or strong, the model predicts "decrease" of the measured species (compared to its level in C1) as early response. Analogously, if all inhibited species act as inhibitors for a measured species, the model prediction for the early differential response of this node is "increase". For the late response, the model predicts "decrease" as the only possible behavior if all inhibited species are strong activators, and "increase" if all inhibited species are strong inhibitors. If all inhibited species are neutral factors, the model prediction is "no change", both for the early and late response. If both positive and negative paths from the inhibited to the measured species exist (either because one of the inhibited species is an ambivalent factor or because one inhibited species is an activator, another one an inhibitor for the measured species) any qualitative early and late response is allowed. Finally, if there is a weak activator or weak inhibitor among the inhibited species, the late response is not restricted by the model.

The effect of some inhibitors can often not be mimicked by perturbing a certain node in the network. The applied MEK inhibitor, for example, blocks MEK kinase activity, thus inhibiting the outgoing edges from MEK. Therefore, we introduce a “dummy” node that is activated by MEK and itself activates all downstream nodes of MEK (in our particular case only ERK). The effect of the MEK inhibitor is then reflected by this dummy node.

The described algorithm was implemented as a MATLAB script making use of API functions of the MATLAB-based toolbox *CellNetAnalyzer* [62], which is freely available for academic use (http://www.mpi-magdeburg.mpg.de/projects/cna/cna.html). The pseudocodes of the algorithm are given in S1 Code. The HGF interaction graph model is provided in *CellNetAnalyzer* format at http://www2.mpi-magdeburg.mpg.de/projects/cna/repository.html.

### Ordinary differential equation modeling

#### Translation of selected minimal interaction graph model structures into ODE models

We can associate an interaction graph with a given system of ODEs: the interaction graph is defined based on the signs of the entries of the Jacobian of the system, which represents the signs of the partial derivatives of the state variables [63]. In this way, one can draw some conclusions on the possible dynamic behavior of the system based on structural properties of the interaction graph [64]. Here, we built for each identified interaction graph substructure a corresponding ODE model, that is, an ODE model whose underlying interaction graph reflects the respective minimal model structure (S5 Fig). A related approach enables the automatic conversion of logical models to ODE systems [65], but cannot be applied here as we utilize interaction graphs in our approach. Here, we built the ODE models from the interaction graphs in the following way: For the core model, each edge pA->pB from the interaction graph, where pA denotes the activated form of a species A and pB the activated form of a species B, gives rise to an ODE reaction describing the formation of pB. With mass action kinetics and assuming pA catalyzes the formation of pB but is not consumed, we get the reaction B->pB with kinetic rate law k1*pA*B, where k1 denotes a kinetic parameter for this reaction. In addition, we introduce the deactivation (for example, dephosphorylation) reaction pB->B with kinetic rate law k2*pB (S4 Table). An exception from this rule is the treatment of the species RSK. The interaction graph model describes activation of RSK as a two-step process: RSK->RSK_s->RSK_d. In this case, we assume that both activated forms (RSK_s and RSK_d) are deactivated to RSK (S4 Table, lines 26–29). For the Met receptor, an additional formation and degradation reaction was necessary (S4 Table, lines 3 and 4) to reflect the observed receptor dynamics. PDK1 is assumed to be constitutively active [66, 67] and was thus included as a constant parameter in the ODE model. To incorporate the effect of inhibitor treatments into the model, inhibitor parameters for PDK1, PI3K, Met and MEK have been introduced. These parameters allow for a potential reduction of the kinetic parameters within a range of 0% to 100%. Furthermore, these parameters are coupled to binary switches, allowing them to be activated only in their respective condition.

For each candidate edge, one additional reaction is added to the ODE model. We distinguish candidate mechanisms that provide an alternative, independent way of activation of the respective species and those that influence the core activation mechanism. An example for the first is the activation of PI3K by Ras, which is described as PI3K -> active PI3K with rate law k*Ras_active*PI3K (S4 Table, line 41). An example for the latter is the effect of PAK on Raf, which is assumed to promote Raf activation through Ras, and which is thus included as Raf-> pRaf with rate law k*Ras_active*pPAK*Raf in the ODE model (S4 Table, line 35). Translation of inhibiting edges of the interaction graph follows the same rules. As an example, deactivation of Raf by Akt is described as pRaf->Raf with rate law k*pAkt*pRaf (S4 Table, line 37).

ODE modeling was performed using a novel MATLAB-based software implementation [68]. Here, we built all model structures in a framework-specific format, which allows import and export to the common data format SBML [69]. The model 4_8_12 is available as a SBML in S1 Model.

#### Parameter estimation

To find the optimal parameter sets that describe the experimental data for each model structure, we performed parameter estimations. The framework is using a parallelised implementation of the CVODES ODE solver [70]. The procedure of parameter estimation is based on multiple local optimizations of different parameter starting values. For the optimizations, the LSQNONLIN algorithm (MATLAB, R2011a, The Mathworks Inc. (Natick, MA)) was used. Parameter values are limited between a range of 0.00001 and 1000, that is, eight orders of magnitude on a logarithmic scale. We assume that this parameter range is sufficient for our model selection approach. Parameter values close to upper or lower boundaries result from partial practical non-identifiability of the model structures. This does not influence the resulting ranking of the model structures. Parameter estimation is based on multiple local optimizations of different parameter starting values. For the random sampling of the starting points, a latin hypercube method is utilized [71]. For each set of randomly selected initial parameter values, a local optimization procedure is performed. Here, parameter estimation of the software has been modified to cope with the high complexity of the parameter estimation problem: As an addition, parameter values that were estimated as being close to their respective upper or lower boundary were automatically fixed for a limited period of optimization cycles. As boundary-close parameters slow down the optimization procedure, temporal removal of these degrees of freedom for a short interval during the process led to an increase in fitting performance. To prove the validity of our optimization procedure, a Latin hypercube sampling approach with 1000 initial parameter sets has been performed (S7 Fig). As shown, the parameter estimation algorithm is able to converge into a global minimum within the parameter landscape in a reproducible manner.

In addition to kinetic parameters, the ODE model is defined by scaling and noise parameters. These non-kinetic parameters were fitted in parallel to the kinetic parameters as described [68].

During the parameter estimation, no steady state conditions have been utilized. We assume that non-stimulated measurements of signalling components in our data set is sufficient for training the system to an initial steady state in an unstimulated setting. Additionally, our ODE model would be able to capture oscillatory behaviour if present.

Model structures feature a variety of negative and positive feedback mechanisms. This allows, in principle, for the occurrence of oscillatory behaviour of signalling components within the considered parameter space. However, as we did not observe oscillations in our experimental data, oscillations are not exhibited by the ODE model.

#### Rankings (AIC/LRT)

Performance of an estimated set of parameter values is characterized by its likelihood *L*. Here, we minimized the negative logarithm of the likelihood function (-2log(*L*)) during the optimization process. Therefore, the best performing model structure in combination with the estimated parameter set is described by the lowest -2log(*L*). To take into account different degrees of freedom of the models, two different methods are common in literature.

First, the Akaike Information Criterion (AIC) is defined as

AIC=2k-2log(L)

where *k* denotes the degrees of freedom in the respective model [72].

The second common method is known as the Likelihood-Ratio-Test (LRT), where one model is defined as the null model and another nested model is compared against the null model [73]. Here, the comparison is performed by

$$icdf_{\Delta df}(0.95)-2\log(L)$$

where Δdf denotes the difference in degrees of freedom of the null model and the nested model. Hereby, one tests if a nested model is a valid simplification of the null model. The complete model is defined as the null model. Each of the tested model structures is therefore compared pairwise against the complete model. The result of each pairwise likelihood ratio test is then used to obtain the ranking of the corresponding model structures. In this work, we present all rankings with unprocessed -2log(*L*) values, AIC ranking and LRT for all model structures against the complete model. While the AIC allows the creation of a complete ranking and therefore a comparison of different candidate models against each other, the LRT provides us with more detailed information regarding the pairwise comparison of a nested model against the null model. In practice, the AIC slightly favours larger models due to the linear penalisation of the degrees of freedom of a model.

#### Predictive power

To compare the predictive power of the selected candidate model 4_8_12 and the complete model, we utilize prediction profiles as described [39]. For our analysis, prediction profiles have been calculated along the complete time course of the selected model 4_8_12 and the complete model for “active PI3K”. Through the calculation of prediction profiles, a range for the specified trajectories of the protein dynamic is given for each calculated time point, in which the likelihood value of the model stays within a 95% threshold.

#### Random models

The selected model structures contain either four or five candidate edges. We decided to consider random models with five candidate edges. Thus, we randomly selected subsets of size five from all candidate edges present in the complete model. The so-derived random models were compared with the minimal model structures: if a random model was identical to or comprised a minimal model structure, this model was rejected. In this way, we generated 50 random models and compared their performance with the minimal model structures (S6 Table and Fig 5D).

#### Simulations

To study the effects of certain protein inhibitions, we performed *in silico* experiments. Here, we down-regulated all outgoing reactions for each protein, one protein at a time, by reducing the respective kinetic parameter to 50% of the original value and simulated the pathway dynamics under this perturbed condition. Furthermore, each possible combination of two protein inhibitions has been simulated. As readout, the area under curve of pAkt, pERK and the sum of pAkt and pERK have been evaluated for the measured time of 120 minutes and compared to the control condition. The corresponding heat maps are shown in Fig 8A).

For the synergy analysis, the efficiency of a double inhibitor treatment and two individual inhibitor treatments is compared (Fig 8B). Positive values mean that the double inhibition condition is having a stronger effect than the two single inhibitions, that is, there is a synergy effect. Negative values mean that the single inhibitions are more effective than the combination. A neutral value of 0 means the two inhibitors work in an additive manner.

## Supporting Information

S1 Table Species in the interaction graph model. The table describes the species present in the interaction graph model. The species are considered in their active form, for example, Akt is considered as Akt phosphorylated on serine 473. Species 11: MEK1/2 phosphorylated at serine 217/221 Species 12: MEK1/2 phosphorylated at serine 298Species 12: MEK1/2 phosphorylated at threonine 292 Species 19, 21, 22: considered in their active GTP-bound form. Species 25, 26: RSK_s refers to p90RSK phosphorylated on a single serine residue. RSK_d refers to p90RSK phosphorylated on two serine residues and considered as active p90RSK.
(DOCX)

S2 Table Mechanisms in the interaction graph model (core model). The table describes the list of reactions of the HGF interaction graph (core model).
(DOCX)

S3 Table Candidate mechanisms in the interaction graph model. The table describes the list of reactions defined as candidate mechanisms in the HGF interaction graph master model. Each number corresponds to a candidate mechanism. The letters indicate the reactions for the candidate mechanisms composed by more than one reaction. The asterisks indicate the edges included in more than one candidate mechanism.
(DOCX)

S4 Table Reactions in the ODE models. The table describes the list of reactions of the ODE models. The first list describes the reactions of the core model (reaction 1–29); the second list describes the reactions of the candidate mechanisms (reaction 30–42). In the second column, each reaction is shown in a schematic representation; in the third column, the respective kinetic rate law is shown. Values of each kinetic parameter for model 4_8_12 are shown in S5 Table. Parameters “Met_inh”, “PDK_inh” and “MEK_inh” represent binary values dependent on the respective experimental condition.
(DOCX)

S5 Table List of parameter names and values of the model 4_8_12. The table describes the list of parameter names and values of the ODE model 4_8_12. The model was calibrated on 2200 data points and 25 experimental conditions. The second column shows the log_10_ value of each kinetic parameter involved in the model reactions shown in S4 Table. The x indicates that the parameter is not present in the model 4_8_12.
(DOCX)

S6 Table List of models and candidate mechanisms. The table describes the list of candidate mechanisms for each selected minimal model structure (gray), model combinations (gray) and random models (rand): 1 indicates that the candidate mechanism is present, 0 indicates that it is not present. The models are sorted according to the Likelihood ratio test value as shown in Fig 5.
(DOCX)

S1 Text References for S1–S6 Tables. (DOCX)

S1 Fig Quantitative immunoblotting. Quantitative immunoblotting of primary mouse hepatocytes treated with MEK inhibitor (U0126), PDK1 inhibitor (BX-912), their combination or DMSO (-) and subsequently stimulated with 40 ng/ml HGF for the indicated time points. A) Immunoprecipitation (IP) of total SOS protein. The band shift indicates the phosphorylated inactive form of SOS (higher band). B) Total cellular lysate was used to detect phosphorylation of MEK1/2, ERK1/2 and Akt on serine 473 and total actin used as normalizer. Quantification of the corresponding immunoblots is shown for pAkt, pMEK, pERK. C) Total cellular lysate was used to detect p90RSK phosphorylation on serine 227 and 380, total p90RSK and protein disulfide-isomerase (PDI) used as normalizer. Quantification of the corresponding immunoblots is shown for p90RSK Ser380 and p90RSK Ser227. Samples were loaded in a random order to reduce the correlated error [60]. Experiments shown in panels B and C were performed at least in triplicates and one representative dataset is shown.
(EPS)

S2 Fig Quantitative immunoblotting of siRNA treatment. A) Quantitative immunoblotting of primary mouse hepatocytes treated with siRNA targeting ERK1/2 and Akt and subsequently stimulated with 40 ng/ml HGF for the indicated time points. Total cellular lysate was used to detect MEK1/2 phosphorylation, total ERK, total MEK1/2 and total actin used as normalizer. Samples of hepatocytes treated with siRNA targeting Akt were processed with the samples of siRNA targeting ERK1/2. Data quantification is shown in S3B Fig. Data of Akt siRNA treatment has not been used for model calibration. Samples were loaded in a random order to reduce the correlated error [60]. B) Knockdown efficiency of siRNA targeting ERK1/2. Signal intensity of total ERK1/2 and actin was measured by LumiAnalysis software (Roche Diagnostic). Total ERK1/2 signal was divided by the respective actin signal and the average of 6 replicas is shown with the corresponding standard deviation. The knockdown efficiency for ERK1/2 siRNA is indicated as percentage of the control condition.
(EPS)

S3 Fig Fold change of protein phosphorylation states. For each protein, the fold change of the phosphorylation state measured by quantitative immunoblotting of two different experimental conditions is shown on a logarithmic scale at the indicated time points after 40 ng/ml of HGF stimulation. A) Fold change of Akt phosphorylation on threonine 308 and the active, not phosphorylated, SOS is shown (representative blot shown in S1A Fig) B) Fold change of the phosphorylation state of the indicated proteins upon PI3K inhibitors treatment. These data resulted to be not conclusive, and, therefore, it has not been discretized and it was not used for model calibration. Each row refers to one experiment; same experimental conditions are grouped with magenta lines.
(EPS)

S4 Fig Predictions from interaction graph structures. Shown are the predictions from all selected minimal model structures (Fig 4) and their comparison with the discretized data shown in Fig 3B. “Early” refers to the first response following HGF stimulation. For later time points, either the first effect that is opposite the initial response is given, or an arrow of same direction indicates that the qualitative response was equal for all time points. Arrow pointing down/up: the inhibition can only cause a decreased/increased activation of the measured protein. Bullet point: the inhibition does not affect the measured protein. Combined up/down arrow and bullet point: the model does not restrict the response to the inhibition.
(EPS)

S5 Fig Underlying interaction graph of the complete ODE model. A) Shown is the interaction graph underlying the complete ODE model, thus representing the sign-structure of its Jacobian matrix. Black arrows indicate positive, red arrows negative influences. Note that PDK1 receives no input and is not consumed in the ODE model. For simplification, it was therefore treated like a parameter, and the edges indicate its influences (partial derivatives) on other components. The same interaction graph would follow if we included PDK1 as a (constant) state variable in the model. Furthermore, in addition to the nodes that are contained in the interaction graph given in Fig 4, which represent the activated forms of the proteins, the underlying interaction graph of the ODE model contains for each protein one node representing its inactive form. With the chosen mass action kinetics (S4 Table), the interaction graph contains for each reaction where the activated form of protein A, pA, positively influences the activation of protein B (B → pB), the positive edge pA → pB and the negative edge pA → B, the latter thus giving rise to an additional indirect negative influence of pA on pB (pA →B → pB). In general, these indirect effects can become visible in the transient dynamics. However, as in our ODE model formulation the active and inactive form of a protein are conserved moieties, the interaction graph of the Jacobian can be reduced to the graph given in panel (B). B) This graph contains the same set of nodes as the interaction graph given in Fig 4, except for an additional node representing the inactive form of the Met receptor: the moieties of Met and pMet are not conserved as additional uptake and degradation is considered (S4 Table). Still, the paths downstream the activated receptor are the same as in the interaction graph in Fig 4. Compared to panel (A), all indirect effects except one could be eliminated: as activation of RSK is modeled as a two-step process, the interaction graph of the ODE model contains with PDK → pRSK_s and pRSK_d → pRSK_d two negative edges that are not contained in the interaction graph in Fig 4. However, even if these edges are considered, the selected substructures of the complete interaction graph model are the minimal structures needed to explain all qualitative effects from the experimental data.
(EPS)

S6 Fig ODE model selection according to the Akaike Information Criterion (AIC). A) Rankings represent the forward selection of selected minimal model structures; B) backward selection, where the building blocks are removed from the complete model; C) the model combinations selection. D) Rankings of model selection including minimal model structures, model combinations and random models. Random model structures consist of randomly selected combinations of candidate edges (S6 Table). Approximately 50% of the random models performed worse than the worst performing selected minimal model structure. Well performing random model structures are closely related to well performing selected minimal model structures and their combination (S6 Table). The Akaike Information Criterion (AIC) has been utilized to penalize the likelihood. All rankings of model selection present the negative logarithmic likelihood penalized by parameter difference on the y-axis. Model identifiers are shown on the x-axis.
(EPS)

S7 Fig Multistart optimization with 1000 initial parameter sets for model 4_8_12. A) Multistart optimization has been performed with 1000 initial parameter sets. Fits have been sorted according to the resulting likelihood. B) Detailed view of panel (A) where the trend of the likelihood of 780 fits is shown.
(EPS)

S8 Fig Comparison of the core model, complete model and model 4_8_12. A) Structure of the core model, the complete model (D), model 4_8_12 (G) and corresponding plots showing representative model trajectories (solid lines) of the phosphorylation kinetic of MEK (B-E-H) and Akt (C-F-I) measured by quantitative immunoblotting in primary mouse hepatocytes pretreated with the indicated inhibitors and stimulated with 40 ng/ml HGF for the indicated time (stars). Values of the likelihood ratio test with the threshold of 95% are shown under each corresponding model. In the plots, y-axes show the concentration of the respective measured protein in arbitrary units on a logarithmic scale. The colored area surrounding the model trajectory represents the confidence interval delimited by the dashed line. Treatments are color-coded as indicated in the figure. The core model (A) does not capture the difference between the experimental conditions while the complete model (D) and model 4_8_12 (G) clearly distinguish them. For example, the complete model and model 4_8_12 capture the effect of MEK inhibitor and PDK1 inhibitor on pMEK1/2 showing a delayed and sustained response.
(EPS)

S9 Fig Negative crosstalk: Experimental validation (replica 1). Quantitative immunoblotting of primary mouse hepatocytes treated with Akt inhibitor VIII or DMSO and subsequently stimulated with 40 ng/ml HGF for the indicated time points. A) Total cellular lysate or immunoprecipitation (IP) was used to detect the indicated proteins. Raf1 protein has a distinguished phosphorylation pattern: Phosphorylation at serine 338 contributes to Raf1 activation, while phosphorylation at serine 259 is an inactivation signal and targeted by Akt (shown in Fig 7). B) Immunoblot data were quantified by ImageQuant TL version 7.0 software (GE Healthcare).
(EPS)

S10 Fig Negative crosstalk: Experimental validation (replica 2). Quantitative immunoblotting of primary mouse hepatocytes treated with Akt inhibitor VIII or DMSO and subsequently stimulated with 40 ng/ml HGF for the indicated time points. A) Total cellular lysate or immunoprecipitation (IP) was used to detect the indicated proteins. Raf1 protein has a distinguished phosphorylation pattern: Phosphorylation at serine 338 contributes to Raf1 activation, while phosphorylation at serine 259 is an inactivation signal and targeted by Akt. B) Immunoblot data were quantified by ImageQuant TL version 7.0 software (GE Healthcare).
(EPS)

S11 Fig Negative crosstalk: Experimental validation (replica 3). Quantitative immunoblotting of primary mouse hepatocytes treated with Akt inhibitor VIII or DMSO and subsequently stimulated with 40 ng/ml HGF for the indicated time points. A) Total cellular lysate or immunoprecipitation (IP) was used to detect the indicated proteins. Raf1 protein has a distinguished phosphorylation pattern: Phosphorylation at serine 338 contributes to Raf1 activation, while phosphorylation at serine 259 is an inactivation signal and targeted by Akt. B) Immunoblot data were quantified by ImageQuant TL version 7.0 software (GE Healthcare).
(EPS)

S12 Fig Inhibitor combination: Experimental validation. Quantitative immunoblotting of primary mouse hepatocytes treated with the inhibitor combinations and 0.1μM Met inhibitor PHA665752 and 10 microM PDK1 inhibitor (BX-912), 0.1 microM P3K inhibitor (PI-103) and 0.1 microM MEK inhibitor (U0126) or DMSO (-) and subsequently stimulated with 40 ng/ml HGF for the indicated time points. A) Total cellular lysate was used to detect phosphorylation of ERK1/2 and Akt on serine 473 and total actin used as normalizer. The quantification plots are shown in Fig 8D. B) Replica of the experimental validation. Total cellular lysate was used to detect phosphorylation of ERK1/2 and Akt on serine 473 and total actin used as normalizer. The plots show the quantification of the corresponding immunoblots.
(EPS)

S1 Code Pseudocodes. Pseudocodes for the selection of minimal model structures from the interaction graph master model.
(PDF)

S1 Model ODE model 4_8_12 formatted in SBML. The 4_8_12 model contains the core edges and all candidate edges deriving from the combination of models 4_8_12.
(XML)

S1 Dataset ExperimentalData_Timecourse_inhibitors_siRNA. The datasets contain the experimental data used for the selection of minimal model structures and model calibration. The datafile is characterized by a header row as follow: Time: duration of HGF stimulation. Units: minutes.Gel: unique number to identify the gel for each analyzed protein. HGF_input: concentration of HGF used for stimulation. Units: ng/ml. Met_ihn: Met inhibitor. Mek_inh: MEK inhibitor. Pdk_inh: PDK1 inhibitor. ERK siRNA: siRNA targeting ERK1/2. pMet_au: Met phosphorylation. Units: arbitrary units (au). pAkt_au: Akt phosphorylation on serine 473. Units: arbitrary units (au). pAktThr_au: Akt phosphorylation on threonine 308. Units: arbitrary units (au). pERK_au: sum of ERK1 and ERK2 phosphorylation. Units: arbitrary units (au). pMEK_au: sum of MEK1 and MEK2 phosphorylation. Units: arbitrary units (au). single_pRSK_au: p90RSK phosphorylation corresponding to the p90RSK_s of the model. Units: arbitrary units (au). double_pRSK_au: p90RSK phosphorylation corresponding to the p90RSK_d of the model. Units: arbitrary units (au). Actin_au: actin. Units: arbitrary units (au). PDI_au: Protein disulfide isomerase. Units: arbitrary units (au). Exp_Gel_ori: experiment identifier is indicated in letters (i.e. “A_tc”); reference number of gel. Gel_ori: internal reference gel number. Exp: internal reference experiment identifier. NaN: no datapoint has been measured for the indicated condition. Columns defining perturbation treatment (inhibitor or siRNA): 0 indicates that the corresponding inhibitor or siRNA was not present; 1 indicates that the corresponding inhibitor or siRNA was present.
(XLSX)

S2 Dataset ExperimentalData_Ras_ProteinArray. The datasets contain the experimental data used for the selection of minimal model structures and model calibration. The datafile is characterized by a header row as follow: Time: duration of HGF stimulation. Units: minutes. Gel: unique number to identify the gel for each analyzed protein. In this special case, “gel” refers to protein array. HGF_input: concentration of HGF used for stimulation. Units: ng/ml Met_ihn: Met inhibitor Mek_inh: MEK inhibitor Pdk_inh: PDK1 inhibitor ERK siRNA: siRNA targeting ERK1/2 Rasnew_au: active Ras measured by protein array. Units: arbitrary units (au) NaN: no datapoint has been measured for the indicated condition. Columns defining perturbation treatment (inhibitor or siRNA): 0 indicates that the corresponding inhibitor or siRNA was not present; 1 indicates that the corresponding inhibitor or siRNA was present.
(XLSX)

S3 Dataset ExperimentalData_Ras_Immunoblot. The datasets contain the experimental data used for the selection of minimal model structures and model calibration. The datafile is characterized by a header row as follow: Time: duration of HGF stimulation. Units: minutes. Gel: unique number to identify the gel for each analyzed protein HGF_input: concentration of HGF used for stimulation. Units: ng/ml Met_ihn: Met inhibitor Mek_inh: MEK inhibitor Pdk_inh: PDK1 inhibitor ERK siRNA: siRNA targeting ERK1/2 aRas_au: active Ras measured by immunoblot. Units: arbitrary units (au) Exp_Gel_ori: experiment identifier is indicated in letters (i.e. “A_tc”); reference number of gel. Gel_ori: internal reference gel number Exp: internal reference experiment identifier.
(XLSX)

S4 Dataset ExperimentalData_ERK phosphorylation_MassSpec. The datasets contain the experimental data used for the selection of minimal model structures and model calibration. The datafile is characterized by a header row as follow: Time: duration of HGF stimulation. Units: minutes. Gel: unique number to identify the gel for each analyzed protein HGF_input: concentration of HGF used for stimulation. Units: ng/ml Met_ihn: Met inhibitor Mek_inh: MEK inhibitor Pdk_inh: PDK1 inhibitor ERK siRNA: siRNA targeting ERK1/2 pERK_au: sum of ERK1 and ERK2 phosphorylation measured by mass spectrometry. Units: arbitrary units (au) Exp_Gel_ori: experiment identifier is indicated in letters (i.e. “A_tc”); reference number of gel. Gel_ori: internal reference gel number Exp: internal reference experiment identifier.
(XLSX)

S5 Dataset ExperimentalData_Akt phosphorylation_MassSpec. The datasets contain the experimental data used for the selection of minimal model structures and model calibration. The datafile is characterized by a header row as follow: Time: duration of HGF stimulation. Units: minutes. Gel: unique number to identify the gel for each analyzed protein HGF_input: concentration of HGF used for stimulation. Units: ng/ml Met_ihn: Met inhibitor Mek_inh: MEK inhibitor Pdk_inh: PDK1 inhibitor ERK siRNA: siRNA targeting ERK1/2 pAkt_rel_obs: Akt phosphorylation on serine 473 measured by mass spectrometry. Units: arbitrary units (au) Akt_rel_obs: unphosphorylated Akt measured by mass spectrometry. Units: arbitrary units (au) Exp_Gel_ori: experiment identifier is indicated in letters (i.e. “A_tc”); reference number of gel.Gel_ori: internal reference gel number Exp: internal reference experiment identifier.
(XLSX)

## References

[1] Pawson T, Warner N. Oncogenic re-wiring of cellular signaling pathways. Oncogene. 2007;26(9):1268–75. 17322911 10.1038/sj.onc.1210255

[2] Borowiak M, Garratt AN, Wustefeld T, Strehle M, Trautwein C, Birchmeier C. Met provides essential signals for liver regeneration. Proc Natl Acad Sci U S A. 2004;101(29):10608–13. 15249655 10.1073/pnas.0403412101 PMC490025

[3] Huh CG, Factor VM, Sanchez A, Uchida K, Conner EA, Thorgeirsson SS. Hepatocyte growth factor/c-met signaling pathway is required for efficient liver regeneration and repair. Proc Natl Acad Sci U S A. 2004;101(13):4477–82. 15070743 10.1073/pnas.0306068101 PMC384772

[4] Douville E, Downward J. EGF induced SOS phosphorylation in PC12 cells involves P90 RSK-2. Oncogene. 1997;15(4):373–83. 9242373 10.1038/sj.onc.1201214

[5] Shin S-Y, Rath O, Choo S-M, Fee F, McFerran B, Kolch W, et al Positive- and negative-feedback regulations coordinate the dynamic behavior of the Ras-Raf-MEK-ERK signal transduction pathway. J Cell Sci. 2009;122(Pt 3):425–35. 10.1242/jcs.036319 19158341

[6] Maroun CR, Holgado-Madruga M, Royal I, Naujokas MA, Fournier TM, Wong AJ, et al The Gab1 PH domain is required for localization of Gab1 at sites of cell-cell contact and epithelial morphogenesis downstream from the met receptor tyrosine kinase. Mol Cell Biol. 1999;19(3):1784–99. 10022866 10.1128/mcb.19.3.1784 PMC83972

[7] Zimmermann S, Moelling K. Phosphorylation and regulation of Raf by Akt (protein kinase B). Science. 1999;286(5445):1741–4. 10576742 10.1126/science.286.5445.1741

[8] Johnson DB, Sosman JA. Update on the targeted therapy of melanoma. Curr Treat Options Oncol. 2013;14(2):280–92. 10.1007/s11864-013-0226-8 23420410 PMC6684217

[9] Goldinger SM, Zimmer L, Schulz C, Ugurel S, Hoeller C, Kaehler KC, et al Upstream mitogen-activated protein kinase (MAPK) pathway inhibition: MEK inhibitor followed by a BRAF inhibitor in advanced melanoma patients. Eur J Cancer. 2014;50(2):406–10. 10.1016/j.ejca.2013.09.014 24183461

[10] Fruman DA, Rommel C. PI3Kdelta inhibitors in cancer: rationale and serendipity merge in the clinic. Cancer Discov. 2011;1(7):562–72. 10.1158/2159-8290.CD-11-0249 22586681

[11] Shapiro GI, Rodon J, Bedell C, Kwak EL, Baselga J, Brana I, et al Phase I safety, pharmacokinetic, and pharmacodynamic study of SAR245408 (XL147), an oral pan-class I PI3K inhibitor, in patients with advanced solid tumors. Clin Cancer Res. 2014;20(1):233–45. 10.1158/1078-0432.CCR-13-1777 24166903

[12] Flinn IW, Kahl BS, Leonard JP, Furman RR, Brown JR, Byrd JC, et al Idelalisib, a selective inhibitor of phosphatidylinositol 3-kinase-delta, as therapy for previously treated indolent non-Hodgkin lymphoma. Blood. 2014;123(22):3406–13. 10.1182/blood-2013-11-538546 24615776 PMC4260978

[13] Hong DS, Bowles DW, Falchook GS, Messersmith WA, George GC, O'Bryant CL, et al A multicenter phase I trial of PX-866, an oral irreversible phosphatidylinositol 3-kinase inhibitor, in patients with advanced solid tumors. Clin Cancer Res. 2012;18(15):4173–82. 10.1158/1078-0432.CCR-12-0714 22693357

[14] Yap TA, Yan L, Patnaik A, Fearen I, Olmos D, Papadopoulos K, et al First-in-man clinical trial of the oral pan-AKT inhibitor MK-2206 in patients with advanced solid tumors. J Clin Oncol. 2011;29(35):4688–95. 10.1200/JCO.2011.35.5263 22025163

[15] Lorusso PM, Adjei AA, Varterasian M, Gadgeel S, Reid J, Mitchell DY, et al Phase I and pharmacodynamic study of the oral MEK inhibitor CI-1040 in patients with advanced malignancies. J Clin Oncol. 2005;23(23):5281–93. 16009947 10.1200/JCO.2005.14.415

[16] LoRusso PM, Krishnamurthi SS, Rinehart JJ, Nabell LM, Malburg L, Chapman PB, et al Phase I pharmacokinetic and pharmacodynamic study of the oral MAPK/ERK kinase inhibitor PD-0325901 in patients with advanced cancers. Clin Cancer Res. 2010;16(6):1924–37. 10.1158/1078-0432.CCR-09-1883 20215549

[17] Rinehart J, Adjei AA, Lorusso PM, Waterhouse D, Hecht JR, Natale RB, et al Multicenter phase II study of the oral MEK inhibitor, CI-1040, in patients with advanced non-small-cell lung, breast, colon, and pancreatic cancer. J Clin Oncol. 2004;22(22):4456–62. 15483017 10.1200/JCO.2004.01.185

[18] Haura EB, Ricart AD, Larson TG, Stella PJ, Bazhenova L, Miller VA, et al A phase II study of PD-0325901, an oral MEK inhibitor, in previously treated patients with advanced non-small cell lung cancer. Clin Cancer Res. 2010;16(8):2450–7. 10.1158/1078-0432.CCR-09-1920 20332327

[19] Grimaldi AM, Cassidy PB, Leachmann S, Ascierto PA. Novel approaches in melanoma prevention and therapy. Cancer Treat Res. 2014;159:443–55. 10.1007/978-3-642-38007-5_25 24114495

[20] Urner-Bloch U, Urner M, Stieger P, Galliker N, Winterton N, Zubel A, et al Transient MEK inhibitor-associated retinopathy in metastatic melanoma. Ann Oncol. 2014;25(7):1437–41. 10.1093/annonc/mdu169 24864047

[21] www.clinicaltrials.gov. Available from: www.clinicaltrials.gov.

[22] Kholodenko BN. Negative feedback and ultrasensitivity can bring about oscillations in the mitogen-activated protein kinase cascades. Eur J Biochem. 2000;267(6):1583–8. 10712587 10.1046/j.1432-1327.2000.01197.x

[23] Schoeberl B, Eichler-Jonsson C, Gilles ED, Muller G. Computational modeling of the dynamics of the MAP kinase cascade activated by surface and internalized EGF receptors. Nat Biotechnol. 2002;20(4):370–5. 11923843 10.1038/nbt0402-370

[24] Klinger B, Sieber A, Fritsche-Guenther R, Witzel F, Berry L, Schumacher D, et al Network quantification of EGFR signaling unveils potential for targeted combination therapy. Mol Syst Biol. 2013;9:673 10.1038/msb.2013.29 23752269 PMC3964313

[25] Borisov N, Aksamitiene E, Kiyatkin A, Legewie S, Berkhout J, Maiwald T, et al Systems-level interactions between insulin-EGF networks amplify mitogenic signaling. Mol Syst Biol. 2009;5:256 10.1038/msb.2009.19 19357636 PMC2683723

[26] Won JK, Yang HW, Shin SY, Lee JH, Heo WD, Cho KH. The crossregulation between ERK and PI3K signaling pathways determines the tumoricidal efficacy of MEK inhibitor. J Mol Cell Biol. 2012;4(3):153–63. 10.1093/jmcb/mjs021 22561840

[27] Santos SD, Verveer PJ, Bastiaens PI. Growth factor-induced MAPK network topology shapes Erk response determining PC-12 cell fate. Nat Cell Biol. 2007;9(3):324–30. 17310240 10.1038/ncb1543

[28] Cirit M, Haugh JM. Data-driven modelling of receptor tyrosine kinase signalling networks quantifies receptor-specific potencies of PI3K- and Ras-dependent ERK activation. Biochem J. 2012;441(1):77–85. 10.1042/BJ20110833 21943356 PMC3687362

[29] Saez-Rodriguez J, Alexopoulos LG, Zhang M, Morris MK, Lauffenburger DA, Sorger PK. Comparing signaling networks between normal and transformed hepatocytes using discrete logical models. Cancer Res. 2011;71(16):5400–11. 10.1158/0008-5472.CAN-10-4453 21742771 PMC3207250

[30] Melas IN, Samaga R, Alexopoulos LG, Klamt S. Detecting and removing inconsistencies between experimental data and signaling network topologies using integer linear programming on interaction graphs. PLoS Comput Biol. 2013;9(9):e1003204 10.1371/journal.pcbi.1003204 24039561 PMC3764019

[31] Laubenbacher R, Stigler B. A computational algebra approach to the reverse engineering of gene regulatory networks. J Theor Biol. 2004;229(4):523–37. 15246788 10.1016/j.jtbi.2004.04.037

[32] Samaga R, Klamt S. Modeling approaches for qualitative and semi-quantitative analysis of cellular signaling networks. Cell Commun Signal. 2013;11(1):43 10.1186/1478-811X-11-43 23803171 PMC3698152

[33] Klamt S, Saez-Rodriguez J, Lindquist JA, Simeoni L, Gilles ED. A methodology for the structural and functional analysis of signaling and regulatory networks. BMC Bioinformatics. 2006;7:56 16464248 10.1186/1471-2105-7-56 PMC1458363

[34] Gebser M, Schaub T, Thiele S, Veber P. Detecting inconsistencies in large biological networks with answer set programming. Theory and Practice of Logic Programming. 2011;11(2–3):37.

[35] Kholodenko BN, Kiyatkin A, Bruggeman FJ, Sontag E, Westerhoff HV, Hoek JB. Untangling the wires: a strategy to trace functional interactions in signaling and gene networks. Proc Natl Acad Sci U S A. 2002;99(20):12841–6. 12242336 10.1073/pnas.192442699 PMC130547

[36] Nelander S, Wang W, Nilsson B, She QB, Pratilas C, Rosen N, et al Models from experiments: combinatorial drug perturbations of cancer cells. Mol Syst Biol. 2008;4:216 10.1038/msb.2008.53 18766176 PMC2564730

[37] Kuepfer L, Peter M, Sauer U, Stelling J. Ensemble modeling for analysis of cell signaling dynamics. Nat Biotechnol. 2007;25(9):1001–6. 17846631 10.1038/nbt1330

[38] Molinelli EJ, Korkut A, Wang W, Miller ML, Gauthier NP, Jing X, et al Perturbation biology: inferring signaling networks in cellular systems. PLoS Comput Biol. 2013;9(12):e1003290 10.1371/journal.pcbi.1003290 24367245 PMC3868523

[39] Kreutz C, Raue A, Timmer J. Likelihood based observability analysis and confidence intervals for predictions of dynamic models. BMC Syst Biol. 2012;6:120 10.1186/1752-0509-6-120 22947028 PMC3490710

[40] Mabuchi S, Ohmichi M, Kimura A, Hisamoto K, Hayakawa J, Nishio Y, et al Inhibition of phosphorylation of BAD and Raf-1 by Akt sensitizes human ovarian cancer cells to paclitaxel. J Biol Chem. 2002;277(36):33490–500. 12087097 10.1074/jbc.M204042200

[41] Amit I, Citri A, Shay T, Lu Y, Katz M, Zhang F, et al A module of negative feedback regulators defines growth factor signaling. Nat Genet. 2007;39(4):503–12. 17322878 10.1038/ng1987

[42] Prasasya RD, Tian D, Kreeger PK. Analysis of cancer signaling networks by systems biology to develop therapies. Semin Cancer Biol. 2011;21(3):200–6. 10.1016/j.semcancer.2011.04.001 21511035

[43] Kummar S, Chen HX, Wright J, Holbeck S, Millin MD, Tomaszewski J, et al Utilizing targeted cancer therapeutic agents in combination: novel approaches and urgent requirements. Nat Rev Drug Discov. 2010;9(11):843–56. 10.1038/nrd3216 21031001

[44] Xu TR, Vyshemirsky V, Gormand A, von Kriegsheim A, Girolami M, Baillie GS, et al Inferring signaling pathway topologies from multiple perturbation measurements of specific biochemical species. Science signaling. 2010;3(113):ra20 10.1126/scisignal.2000517 20234003

[45] Corbalan-Garcia S, Yang SS, Degenhardt KR, Bar-Sagi D. Identification of the mitogen-activated protein kinase phosphorylation sites on human Sos1 that regulate interaction with Grb2. Mol Cell Biol. 1996;16(10):5674–82. 8816480 10.1128/mcb.16.10.5674 PMC231567

[46] Wang Z, Wang M, Carr BI. Hepatocyte growth factor enhances protein phosphatase Cdc25A inhibitor compound 5-induced hepatoma cell growth inhibition via Akt-mediated MAPK pathway. J Cell Physiol. 2005;203(3):510–9. 15534860 10.1002/jcp.20243

[47] Romano D, Nguyen LK, Matallanas D, Halasz M, Doherty C, Kholodenko BN, et al Protein interaction switches coordinate Raf-1 and MST2/Hippo signalling. Nat Cell Biol. 2014;16(7):673–84. 10.1038/ncb2986 24929361

[48] Kobayashi T, Aoki Y, Niihori T, Cave H, Verloes A, Okamoto N, et al Molecular and clinical analysis of RAF1 in Noonan syndrome and related disorders: dephosphorylation of serine 259 as the essential mechanism for mutant activation. Hum Mutat. 2010;31(3):284–94. 10.1002/humu.21187 20052757

[49] Ferrell JE Jr., Machleder EM. The biochemical basis of an all-or-none cell fate switch in Xenopus oocytes. Science. 1998;280(5365):895–8. 9572732 10.1126/science.280.5365.895

[50] Soule C. Graphic requirements for multistationarity. Complex Us. 2003(1):10.

[51] Tyson JJ, Chen KC, Novak B. Sniffers, buzzers, toggles and blinkers: dynamics of regulatory and signaling pathways in the cell. Curr Opin Cell Biol. 2003;15(2):221–31. 12648679 10.1016/s0955-0674(03)00017-6

[52] Angeli D, Hirsch MW, Sontag ED. Attractors in coherent systems of differential equations. Journal Differential Equations 2009(246):18.

[53] Brandman O, Meyer T. Feedback loops shape cellular signals in space and time. Science. 2008;322(5900):390–5. 10.1126/science.1160617 18927383 PMC2680159

[54] Meyer T, Stryer L. Molecular model for receptor-stimulated calcium spiking. Proc Natl Acad Sci U S A. 1988;85(14):5051–5. 2455890 10.1073/pnas.85.14.5051 PMC281685

[55] Bhalla US, Ram PT, Iyengar R. MAP kinase phosphatase as a locus of flexibility in a mitogen-activated protein kinase signaling network. Science. 2002;297(5583):1018–23. 12169734 10.1126/science.1068873

[56] Dhillon AS, Hagan S, Rath O, Kolch W. MAP kinase signalling pathways in cancer. Oncogene. 2007;26(22):3279–90. 17496922 10.1038/sj.onc.1210421

[57] Fruman DA, Rommel C. PI3K and cancer: lessons, challenges and opportunities. Nature reviews Drug discovery. 2014;13(2):140–56. 10.1038/nrd4204 24481312 PMC3994981

[58] Huard J, Mueller S, Gilles ED, Klingmuller U, Klamt S. An integrative model links multiple inputs and signaling pathways to the onset of DNA synthesis in hepatocytes. FEBS J. 2012;279(18):3290–313. 10.1111/j.1742-4658.2012.08572.x 22443451 PMC3466406

[59] Hahn B, D'Alessandro LA, Depner S, Waldow K, Boehm ME, Bachmann J, et al Cellular ERK phospho-form profiles with conserved preference for a switch-like pattern. J Proteome Res. 2013;12(2):637–46. 10.1021/pr3007232 23210697

[60] Schilling M, Maiwald T, Bohl S, Kollmann M, Kreutz C, Timmer J, et al Computational processing and error reduction strategies for standardized quantitative data in biological networks. FEBS J. 2005;272(24):6400–11. 16336276 10.1111/j.1742-4658.2005.05037.x

[61] Meyer R, D'Alessandro LA, Kar S, Kramer B, She B, Kaschek D, et al Heterogeneous kinetics of AKT signaling in individual cells are accounted for by variable protein concentration. Front Physiol. 2012;3:451 10.3389/fphys.2012.00451 23226133 PMC3508424

[62] Klamt S, Saez-Rodriguez J, Gilles ED. Structural and functional analysis of cellular networks with CellNetAnalyzer. BMC Syst Biol. 2007;1:2 17408509 10.1186/1752-0509-1-2 PMC1847467

[63] Thieffry D. Dynamical roles of biological regulatory circuits. Brief Bioinform. 2007;8(4):220–5. 17626067 10.1093/bib/bbm028

[64] Radde N, Bar N.S., Banaji M. Graphical methods for analysing feedback in biological networks—A survey. Int J Syst Sci. 2010;41:35–46.

[65] Krumsiek J, Polsterl S, Wittmann DM, Theis FJ. Odefy—from discrete to continuous models. BMC Bioinformatics. 2010;11:233 10.1186/1471-2105-11-233 20459647 PMC2873544

[66] Wick MJ, Wick KR, Chen H, He H, Dong LQ, Quon MJ, et al Substitution of the autophosphorylation site Thr516 with a negatively charged residue confers constitutive activity to mouse 3-phosphoinositide-dependent protein kinase-1 in cells. J Biol Chem. 2002;277(19):16632–8. 11877406 10.1074/jbc.M112402200

[67] Casamayor A, Morrice NA, Alessi DR. Phosphorylation of Ser-241 is essential for the activity of 3-phosphoinositide-dependent protein kinase-1: identification of five sites of phosphorylation in vivo. Biochem J. 1999;342 (Pt 2):287–92. 10455013 PMC1220463

[68] Raue A, Schilling M, Bachmann J, Matteson A, Schelke M, Kaschek D, et al Lessons learned from quantitative dynamical modeling in systems biology. PLoS One. 2013;8(9):e74335 10.1371/journal.pone.0074335 24098642 PMC3787051

[69] Hucka M, Finney A, Sauro HM, Bolouri H, Doyle JC, Kitano H, et al The systems biology markup language (SBML): a medium for representation and exchange of biochemical network models. Bioinformatics. 2003;19(4):524–31. 12611808 10.1093/bioinformatics/btg015

[70] Hindmarsh AC, Brown PN, Grant KE, Lee SL, Serban R, Shumaker DE, et al SUNDIALS: Suite of nonlinear and differential/algebraic equation solvers. ACM Transactions on Mathematical Software (TOMS)—Special issue on the Advanced CompuTational Software (ACTS) Collection 2005;31(3):33.

[71] Owen AB. Orthogonal arrays for computer experiments, integration and visualization. Statistica Sinica. 1992;2:13.

[72] Akaike H. A new look at the statistical model identification. Automatic Control, IEEE Transactions on. 1974:8.

[73] Chernoff H, Lehmann EL. The Use of Maximum Likelihood Estimates in χ2 Tests for Goodness of Fit. The Annals of Mathematical Statistics. 1954;25(3):8.

