# Supplementary material for: Disentangling the Complexity of HGF Signaling by Combining Qualitative and Quantitative Modeling
Source: PLoS Comput Biol. 2015 Apr 23;11(4):e1004192. doi: 10.1371/journal.pcbi.1004192 (PMC4427303; doi:10.1371/journal.pcbi.1004192)
Supplement: S1 Table — The table describes the species present in the interaction graph model. The species are considered in their active form, for example, Akt is considered as Akt phosphorylated on serine 473. Species 11: MEK1/2 phosphorylated at serine 217/221 Species 12: MEK1/2 phosphorylated at serine 298Species 12: MEK1/2 phosphorylated at threonine 292 Species 19, 21, 22: considered in their active GTP-bound form. Species 25, 26: RSK_s refers to p90RSK phosphorylated on a single serine residue. RSK_d refers to p90RSK phosphorylated on two serine residues and considered as active p90RSK. (DOCX) [file pcbi.1004192.s001.docx]

**S1 Table: Species in the interaction graph model**

| \| **Nr.** \| **Model Name** \| **Full Name** \| \| --- \| --- \| --- \| \| 1 \| Akt \|  \| \| 2 \| BRaf \|  \| \| 3 \| crk_CRKL \| c-Crk or Crk-like \| \| 4 \| c-Src \|  \| \| 5 \| C3G \| guanine nucleotide-releasing protein \| \| 6 \| Dock180 \| dedicator of cytokinesis \| \| 7 \| ERK \| extracellular signal-regulated kinase 1 (ERK1) or 2 (ERK2) \| \| 8 \| Gab1 \| Grb2-associated binding protein 1 \| \| 9 \| Grb2 \| growth factor receptor-bound protein 2 \| \| 10 \| HGF \| hepatocyte growth factor \| \| 11 \| MEK \| MAPK/ERK kinase 1 (MEK1) or 2 (MEK2) \| \| 12 \| MEK_S298 \| \| 13 \| MEK_T292 \| \| 14 \| Met \|  \| \| 15 \| PAK \| p21 protein (Cdc42/Rac)-activated kinase \| \| 16 \| PDK1 \| 3-phosphoinositide-dependent protein kinase-1 \| \| 17 \| PIP3 \| phosphatidylinositol (3,4,5)-triphosphate (PI(3,4,5)P_3_) \| \| 18 \| PI3K \| phosphatidylinositol-4,5.bisphosphate 3-kinase \| \| 19 \| Rac \|  \| \| 20 \| Raf1 \|  \| \| 21 \| Rap1 \| Ras-associated protein-1 \| \| 22 \| Ras \| Rat sarcoma \| \| 23 \| RasGAP \| Ras GTPase activating protein \| \| 24 \| RKIP \| Raf-1 kinase inhibitor protein \| \| 25 \| RSK_s \| 90 kDa ribosomal S6 kinase \| \| 26 \| RSK_d \| \| 27 \| SHC \| Src homology 2 domain containing transforming protein 1 \| \| 28 \| SHP2 \| Src homology 2 domain containing phosphotyrosine phosphatase \| \| 29 \| SOS1 \| son of sevenless homolog 1 \| \| 30 \| SOS1_Eps8_E3b1 \| complex of SOS1, Eps8, and E3b1/Abi-1 \| |
| --- | --- | --- | --- | --- | --- | --- | --- | --- | --- | --- | --- | --- | --- | --- | --- | --- | --- | --- | --- | --- | --- | --- | --- | --- | --- | --- | --- | --- | --- | --- | --- | --- | --- | --- | --- | --- | --- | --- | --- | --- | --- | --- | --- | --- | --- | --- | --- | --- | --- | --- | --- | --- | --- | --- | --- | --- | --- | --- | --- | --- | --- | --- | --- | --- | --- | --- | --- | --- | --- | --- | --- | --- | --- | --- | --- | --- | --- | --- | --- | --- | --- | --- | --- | --- | --- | --- | --- | --- | --- | --- |

**S1 Table.** The table describes the species present in the interaction graph model. The species are considered in their active form, for example Akt is considered as Akt phosphorylated on serine 473.

Species 11: MEK1/2 phosphorylated at serine 217/221

Species 12: MEK1/2 phosphorylated at serine 298

Species 12: MEK1/2 phosphorylated at threonine 292

Species 19, 21, 22: considered in their active GTP-bound form.

Species 25, 26: RSK_s refers to p90RSK phosphorylated on a single serine residue. RSK_d refers to p90RSK phosphorylated on two serine residues and considered as active p90RSK.
