# Supplementary material for: Disentangling the Complexity of HGF Signaling by Combining Qualitative and Quantitative Modeling
Source: PLoS Comput Biol. 2015 Apr 23;11(4):e1004192. doi: 10.1371/journal.pcbi.1004192 (PMC4427303; doi:10.1371/journal.pcbi.1004192)
Supplement: S2 Table — The table describes the list of reactions of the HGF interaction graph (core model). (DOCX) [file pcbi.1004192.s002.docx]

**S2 Table: Mechanisms in the interaction graph model (core model).**

| **Activation of Met and adaptor proteins** | | |
| --- | --- | --- |
|  | **Reaction** | **Notes** |
| 1 | HGF → Met | Binding of HGF to Met induces autophosphorylation of the receptor at tyrosines 1334 and 1335[[1](#_ENREF_1)]. Subequently Met phosphorylation occurs at tyrosines 1349 and 1356 (Y1349 and Y1356 respectively). |
| 2 | Met → SHC | The PTB of SHC1 associates to Y1349 and Y1356 of Met [[1](#_ENREF_1)]. |
| 3 | Met → Grb2 | Grb2 is recruited to Met directly (Y1356) and indirectly via SHC1 [[1](#_ENREF_1)]. |
| 4 | SHC → Grb2 |  |
| 5 | Met → cSrc | The SH2 domain of cSrc can bind both Y1349 and Y1356 of Met [[2](#_ENREF_2)]. |
| 6 | Met → Gab1 | Gab1 binds Met both directly (Y1349) and indirectly via a Grb2-binding at Y1356 [[1](#_ENREF_1)]. |
| 7 | Grb2 → Gab1 |  |
| 8 | c-Src → Gab1 | c-Src binds to and phosphorylates Gab1 in response to HGF. Inhibiting c-Src partially inhibited HGF-induced Gab1 phosphorylation by 40–60% [[3](#_ENREF_3)]. Additional phosphorylation through Met is required for Gab1 activation. One hypothesis is that c-Src and Met predominantly phosphorylate Gab1 at different tyrosine residues [[3](#_ENREF_3)]. |
| 9 | Gab1 → Crk_CRKL | c-Crk/CRKL binds to Gab1 after HGF stimulation [[4](#_ENREF_4),[5](#_ENREF_5)]. |
|  | | |
| **Activation of G-proteins Ras and Rac** | | |
|  | **Reaction** | **Notes** |
| 10 | Grb2 → SOS1 | SOS1 is bound to Grb2. Recruitment of Grb2 to the plasma membrane leads to activation of SOS1. |
| 11 | SOS1 → Ras | Interaction of Ras with SOS1 increases the rate of GDP/GTP exchange of Ras [[6](#_ENREF_6)]. |
| 12 | Crk_CRKL → Dock180 | Coexpression of Crk with Dock180 results in the activation of the JNK pathway, indicating that Dock180 connects Crk to the Rac/JNK pathway. However, the possibility that Crk acts through SOS1 cannot be ruled out [[7](#_ENREF_7)]. |
| 13 | Dock180 → Rac | Dock180 is a Rac-specific GEF [[8](#_ENREF_8)]. |
| **Activation of PI3K pathway** | | |
|  | **Reaction** | **Notes** |
| 14 | Met → PI3K | PI3K can bind to Met directly[[5](#_ENREF_5)] and indirectly via Gab1 [[1](#_ENREF_1)]. |
| 15 | Gab1 → PI3K |  |
| 16 | PI3K → PIP3 | PI3K phosphorylates PI(4,5)P_2_ at the D3 position and thus generates PI(3,4,5)P_3_ [[9](#_ENREF_9)]. Since PI(4,5)P_2_ is one of the major phosphorylated forms of PtdIns [[10](#_ENREF_10)], we assume that it is always present in the cell and do not consider its regulation. |
| 17 | PIP3 → Akt | PI(3,4,5)P_3_ recruits Akt and PDK1 to the plasma membrane. Akt is subsequently phosphorylated on serine 473 and threonine 308 [[11](#_ENREF_11)]. |
| 18 | PIP3 → Akt |  |

| **Activation of MAPK pathway** | | |
| --- | --- | --- |
|  | **Reaction** | **Notes** |
| 19 | Ras → Raf1 | Ras recruits Raf1 to the plasma membrane where it is phosphorylated at various sites [[12](#_ENREF_12)]. |
| 20 | Raf1 → MEK | MEK1/2 can be activated by Raf1 [[13](#_ENREF_13)]. |
| 21 | MEK → ERK | MEK1/2 phosphorylate ERK1/2 [[14](#_ENREF_14)]. |
| 22 | ERK → RSK_s | ERK1/2 and PDK1 phosphorylate p90RSK. ERK1/2 activates the C-terminal domain, PDK1 the N-terminal domain, whereas the first is necessary for the latter [[15](#_ENREF_15)]. |
| 23 | PDK1 → RSK_d |  |
| 24 | RSK_s → RSK_d |  |
| 25 | Grb2 → PAK | PAK is recruited to the plasma membrane via Grb2 [[16](#_ENREF_16)], where it is activated through GTP-bound Rac [[17](#_ENREF_17)]. |
| 26 | Rac → PAK |  |

**S2 Table.**

The table describes the list of reactions of the HGF interaction graph (core model).
