# Supplementary material for: Disentangling the Complexity of HGF Signaling by Combining Qualitative and Quantitative Modeling
Source: PLoS Comput Biol. 2015 Apr 23;11(4):e1004192. doi: 10.1371/journal.pcbi.1004192 (PMC4427303; doi:10.1371/journal.pcbi.1004192)
Supplement: S3 Table — The table describes the list of reactions defined as candidate mechanisms in the HGF interaction graph master model. Each number corresponds to a candidate mechanism. The letters indicate the reactions for the candidate mechanisms composed by more than one reaction. The asterisks indicate the edges included in more than one candidate mechanism. (DOCX) [file pcbi.1004192.s003.docx]

**S3 Table: Candidate mechanisms in the interaction graph model.**

| **Nr.** | **Reaction** | **Notes** |
| --- | --- | --- |
| 1a | Gab1 → RasGAP | RasGAP binds phosphorylated Gab1[[19](#_ENREF_19)]. |
| 1b* | RasGAP —\| Ras | The GTPase activating protein RasGAP induces the hydrolysis of Ras-bound GTP to GDP [[20](#_ENREF_20)]. |
| 2a | Gab1 → SHP2 | SHP2 binds to phosphorylated Gab1 in response to HGF stimulation [[21](#_ENREF_21)]. |
| 2b | SHP2 —\| RasGAP | SHP2 dephosphorylates the RasGAP binding site on Gab1 [[22](#_ENREF_22)]. |
| 2c* | RasGAP —\| Ras | The GTPase activating protein RasGAP induces the hydrolysis of Ras-bound GTP to GDP [[20](#_ENREF_20)]. |
| 3a | Crk_CRKL → C3G | Crk and CRKL affect the Rap1 activator C3G. C3G-dependent Rap1 activation as well as CRKL recruitment to Gab1 has been shown in response to HGF [[5](#_ENREF_5)]. |
| 3b | C3G → Rap1 |  |
| 3c | Rap1 → BRaf | Rap1 activates BRaf in different cell types and for different stimuli[[23](#_ENREF_23)]. In human melanoma cell lines, Rap1-dependent activation of ERK in response to HGF was shown[[24](#_ENREF_24)]. |
| 3d | BRaf → MEK | MEK1/2 can be activated by Raf1 or BRaf [[14](#_ENREF_14)]. |
| 4 | ERK —\| SOS1 | Serine/threonine phosphorylation of SOS1 by ERK1/2 and/or p90RSK causes dissociation of Grb2-SOS1 [[25](#_ENREF_25)]. |
| 5 | RSK_d —\| SOS1 |  |
| 6a | ERK —\| RKIP | ERK-mediated phosphorylation of RKIP triggers RKIP dissociation from Raf1 and thus counteracts the inhibitory function of RKIP [[26](#_ENREF_26)]. |
| 6b | RKIP —\| Raf1 | RKIP association with Raf1 disrupts the interaction between MEK1 and Raf1, which is required for MEK1phosphorylation [[27](#_ENREF_27)]. |
| 7 | ERK —\| Raf1 | ERK phosphorylates Raf1 at five inhibitory serine sites [[28](#_ENREF_28)]. |
| 8 | PDK1 → MEK | PDK1 phosphorylates MEK1 on serine222 and MEK2 onserine 226 (MEK2) [[28](#_ENREF_28),[29](#_ENREF_29)]. |
| 9a | PIP3 → SOS1_Eps8_E3b1 | SOS1, Eps8 and E3b1/Abi-1 form a complex which is necessary for the Rac-GEF activity of SOS1. The basal Rac-GEF activity is increased by PI(3,4,5)P_3_ [[30](#_ENREF_30),[31](#_ENREF_31)]. |
| 9b | SOS1_Eps8_E3b1 → Rac |  |
| 10 | PAK → Raf1 | PAK phosphorylates Raf1on serine 338 [[13](#_ENREF_13)]. |
| 11a | PAK → MEK_S298 | PAK phosphorylates MEK1, increasing the ability of Raf1 to activate MEK1 [[32](#_ENREF_32)]. |
| 11b* | MEK_S298 → MEK |  |
| 12a | ERK → MEK_T292 | ERK phosphorylates MEK1 on threonine 292, preventing further phosphorylation of MEK1 by PAK on serine 298 [[32](#_ENREF_32)]. |
| 12b | MEK_T292 —\| MEK_S298 |  |
| 12c* | MEK_S298 → MEK |  |
| 13 | PAK → Akt | PAK promotes Akt recruitment to the plasma membrane and functions as a scaffold to facilitate complex formation between PDK1 and Akt [[33](#_ENREF_33)]. |
| 14 | ERK → PI3K | ERK phosphorylates Gab1 in response to HGF and potentiates Gab1-PI3K association and subsequent PI3K activation [[34](#_ENREF_34)]. |
| 15 | Ras → PI3K | GTP-bound Ras activates PI3K [[35](#_ENREF_35)]. |
| 16 | PIP3 → Gab1 | Plasma membrane localization of Gab1 requires PI(3,4,5)P_3_ [[21](#_ENREF_21),[36](#_ENREF_36)] |
| 17 | Akt —\| Raf1 | Akt negatively regulates Raf1 by phosphorylation of serine 259 [[37](#_ENREF_37),[38](#_ENREF_38)]. |

**S3 Table.**

The table describes the list of reactions defined as candidate mechanisms in the HGF interaction graph master model.

Each number corresponds to a candidate mechanism. The letters indicates the reactions for the candidate mechanisms composed by more than one reaction. The asterisks indicate the edges included in more than one candidate mechanism.
