# Supplementary material for: Disentangling the Complexity of HGF Signaling by Combining Qualitative and Quantitative Modeling
Source: PLoS Comput Biol. 2015 Apr 23;11(4):e1004192. doi: 10.1371/journal.pcbi.1004192 (PMC4427303; doi:10.1371/journal.pcbi.1004192)
Supplement: S4 Table — The table describes the list of reactions of the ODE models. The first list describes the reactions of the core model (reaction 1–29); the second list describes the reactions of the candidate mechanisms (reaction 30–42). In the second column, each reaction is shown in a schematic representation; in the third column, the respective kinetic rate law is shown. Values of each kinetic parameter for model 4_8_12 are shown in S5 Table. Parameters “Met_inh”, “PDK_inh” and “MEK_inh” represent binary values dependent on the respective experimental condition. (DOCX) [file pcbi.1004192.s004.docx]

**S4 Table: Reactions in the ODE models.**

| **Nr.** | **Reactions core model** | **Kinetic rate law** |
| --- | --- | --- |
| 1 | Met receptor → Phosphorylated Met receptor | Met_activation * Met * HGF / (1 + Met_act_inh*Met_inh) |
| 2 | Phosphorylated Met receptor → Met receptor | pMet_dephosphorylation * pMet |
| 3 | Ø → Met receptor | Met_prod_deg_ss |
| 4 | Met receptor → Ø | Met * Met_prod_deg_ss |
| 5 | Phosphorylated Met receptor → Ø | pMet_degradation * pMet |
| 6 | Gab1 + Phosphorylated Met receptor → Gab1_Met receptor complex | Gab1_pMet_binding * Gab1 * pMet |
| 7 | Gab1_Met receptor complex → Gab1 + Phosphorylated Met receptor | Gab1_pMet_diss * Gab1_pMet |
| 8 | Rac → Active Rac | Rac_activation * Rac * Gab1_pMet |
| 9 | Active Rac → Rac | Rac_deactivation * Rac_active |
| 10 | Pak → Phosphorylated Pak | PAK_phosphorylation * PAK1 * Rac_active |
| 11 | Phosphorylated Pak → Pak | PAK_dephosphorylation * pPAK1 |
| 12 | PI3K → Active PI3K | PI3K_activation_by_Gab1 * PI3K * Gab1_pMet |
| 13 | Active PI3K → PI3K | PI3K_inactivation * PI3K_active |
| 14 | Akt → Phosphorylated Akt | Akt_activation_supported_by_PDK1 * Akt * PI3K_active / (1 + PDK_act_inh*PDK_inh) |
| 15 | Phosphorylated Akt → Akt | pAkt_deactivation * pAkt |
| 16 | SOS → SOS_Met receptor complex | SOS_recruitment_by_pMet * SOS * pMet |
| 17 | SOS_Met receptor complex → SOS | mSOS_release_from_membrane * mSOS_pMet |
| 18 | Ras → Active Ras | Ras_activation_by_mSOS * Ras * mSOS_pMet |
| 19 | Active Ras → Ras | Ras_deactivation * Ras_active |
| 20 | Raf → Phosphorylated Raf | Raf_activation * Raf * Ras_active |
| 21 | Phosphorylated Raf → Raf | Raf_inactivation * pRaf |
| 22 | MEK → Phosphorylated MEK | MEK_phosphorylation_by_pRaf * MEK * pRaf |
| 23 | Phosphorylated MEK → MEK | MEK_dephosphorylation * pMEK |
| 24 | ERK → Phosphorylated ERK | ERK_phosphorylation_by_pMEK * ERK * pMEK / (1 + MEK_act_inh*MEK_inh) |
| 25 | Phosphorylated ERK → ERK | ERK_dephosphorylation * pERK |
| 26 | RSK → Single phosphorylated RSK | RSK_phosphorylation_by_pERK*pERK*RSK |
| 27 | Single phosphorylated RSK → Double phosphorylated RSK | RSK_phosphorylation_by_PDK1*single_pRSK / (1 + PDK_act_inh*PDK_inh) |
| 28 | Single phosphorylated RSK → RSK | RSK_dephosphorylation_single * single_pRSK |
| 29 | Double phosphorylated RSK → RSK | RSK_dephosphorylation_double * double_pRSK |

| **Nr.** | **Reactions candidate mechanisms** | **Kinetic rate law** |
| --- | --- | --- |
| 30 | Gab1 + Phosphorylated Met receptor → Gab1_Met receptor complex | Gab1_pMet_binding_by_PI3K_active * Gab1 * pMet * PI3K_active |
| 31 | Rac → Active Rac | Rac_activation_by_PI3k_active * Rac * PI3K_active * Gab1_pMet |
| 32 | SOS_Met receptor complex → SOS | mSOS_release_by_pERK * mSOS_pMet * pERK |
| 33 | SOS_Met receptor complex → SOS | mSOS_release_by_pRSK * mSOS_pMet * double_pRSK |
| 34 | Ras → Active Ras | Ras_activation_by_Gab1 * Ras * Gab1_pMet * mSOS_pMet |
| 35 | Raf → Phosphorylated Raf | Raf_activation_by_PAK * Raf * pPAK1 * Ras_active |
| 36 | Raf → Phosphorylated Raf | Raf_activation_by_pERK * Raf * pERK * Ras_active |
| 37 | Phosphorylated Raf → Raf | pRaf_dephosphorylation_by_Akt * pRaf * pAkt |
| 38 | MEK → Phosphorylated MEK | MEK_phosphorylation_by_PDK1 * MEK * pRaf / (1 + PDK_act_inh*PDK_inh) |
| 39 | MEK → Phosphorylated MEK | MEK_phosphorylation_by_Gab1 * MEK * Gab1_pMet |
| 40 | Mek → Phosphorylated MEK | MEK_phosphorylation_by_pPAK * MEK * pPAK1 * pRaf |
| 41 | PI3K → Active PI3K | PI3K_activation_by_Ras_active * PI3K * Ras_active |
| 42 | PI3K → Active PI3K | PI3K_activation_by_pERK * PI3K * pERK * Gab1_pMet |

**S4 Table.**

The table describes the list of reactions of the ODE models. The first list describes the reactions of the core model (reaction 1-29); the second list describes the reactions of the candidate mechanisms (reaction 30-42). In the second column, each reaction is shown in a schematic representation; in the third column, the respective kinetic rate law is shown. Values of each kinetic parameter for model 4-8-12 are shown in S5 Table. Parameters “Met_inh”, “PDK_inh” and “MEK_inh” represent binary values dependent on the respective experimental condition.
