# Supplementary material for: Disentangling the Complexity of HGF Signaling by Combining Qualitative and Quantitative Modeling
Source: PLoS Comput Biol. 2015 Apr 23;11(4):e1004192. doi: 10.1371/journal.pcbi.1004192 (PMC4427303; doi:10.1371/journal.pcbi.1004192)
Supplement: S5 Table — The table describes the list of parameter names and values of the ODE model 4_8_12. The model was calibrated on 2200 data points and 25 experimental conditions. The second column shows the log10 value of each kinetic parameter involved in the model reactions shown in S4 Table. The x indicates that the parameter is not present in the model 4_8_12. (DOCX) [file pcbi.1004192.s005.docx]

**S5 Table: List of parameter names and values of the model 4_8_12.**

| **Parameter Name** | **Estimated Parameter Value (log_10_) of the model 4_8_12** |
| --- | --- |
| 'Akt_activation_supported_by_PDK1' | -0.27659584 |
| 'ERK_dephosphorylation' | -0.384157706 |
| 'ERK_down' | 1.194738824 |
| 'ERK_phosphorylation_by_pMEK' | 2.982496902 |
| 'Gab1_pMet_binding' | -4.601296578 |
| 'Gab1_pMet_binding_by_PI3K_active' | -0.409307235 |
| 'Gab1_pMet_diss' | -1.488874084 |
| 'MEK_dephosphorylation' | 2.79250161 |
| 'MEK_phosphorylation_by_PDK1' | 2.999999786 |
| 'MEK_phosphorylation_by_pRaf' | -1.999156196 |
| 'Mek_act_inh' | 1.666481986 |
| 'Met_act_inh' | 2.54026603 |
| 'Met_activation' | -1.934829524 |
| 'Met_prod_deg_ss' | -2.89724024 |
| 'PAK_dephosphorylation' | -4.944639884 |
| 'PAK_phosphorylation' | 1.778987643 |
| 'PI3K_activation_by_Gab1' | -4.945663945 |
| 'PI3K_activation_by_Ras_active' | 1.887411595 |
| 'PI3K_activation_by_pERK' | 0.445509919 |
| 'PI3K_inactivation' | 0.159466716 |
| 'Pdk_act_inh' | 0.196825209 |
| 'RSK_dephosphorylation_double' | 0.787276371 |
| 'RSK_dephosphorylation_single' | -0.7166527 |
| 'RSK_phosphorylation_by_PDK1' | -0.568280334 |
| 'RSK_phosphorylation_by_pERK' | -0.559320897 |
| 'Rac_activation' | -4.963891394 |
| 'Rac_deactivation' | -4.999447793 |
| 'Raf_activation' | -0.834642816 |
| 'Raf_activation_by_PAK' | 0.512776283 |
| 'Raf_activation_by_pERK' | 0.223987013 |
| 'Raf_inactivation' | 0.687062866 |
| 'Ras_activation_by_mSOS' | 2.552300571 |
| 'Ras_deactivation' | -1.159750752 |
| 'SOS_recruitment_by_pMet' | -3.42126982 |
| 'mSOS_release_by_pRSK' | 3 |
| 'mSOS_release_from_membrane' | -4.999999297 |
| 'pAkt_deactivation' | 0.348215544 |
| 'pMet_degradation' | -0.86658565 |
| 'pMet_dephosphorylation' | -0.302018341 |
| 'pRaf_dephosphorylation_by_Akt' | 3 |
| 'MEK_phosphorylation_by_Gab1' | x |
| 'MEK_phosphorylation_by_pPAK' | x |
| 'Rac_activation_by_PI3k_active' | x |
| 'Ras_activation_by_Gab1' | x |
| 'mSOS_release_by_pERK' | x |

**S5 Table**

The table describes the list of parameter names and values of the ODE model 4_8_12. The model was calibrated on 2200 data points and 25 experimental conditions. The second column shows the log_10_ value of each kinetic parameter involved in the model reactions shown in S4 Table. The x indicates that the parameter is not present in the model 4_8_12.
