# Supplementary material for: Disentangling the Complexity of HGF Signaling by Combining Qualitative and Quantitative Modeling
Source: PLoS Comput Biol. 2015 Apr 23;11(4):e1004192. doi: 10.1371/journal.pcbi.1004192 (PMC4427303; doi:10.1371/journal.pcbi.1004192)
Supplement: S1 Text — (DOCX) [file pcbi.1004192.s007.docx]

**Supporting references for S1-S6 Tables**

1. Furge KA, Zhang YW, Woude GFV. Met receptor tyrosine kinase: enhanced signaling through adapter proteins. Oncogene. 2000;19(49):5582-9.

2. Ponzetto C, Bardelli A, Zhen Z, Maina F, dalla Zonca P, Giordano S, et al. A multifunctional docking site mediates signaling and transformation by the hepatocyte growth factor/scatter factor receptor family. Cell. 1994;77(2):261-71.

3. Chan P-C, Chen Y-L, Cheng C-H, Yu K-C, Cary LA, Shu K-H, et al. Src phosphorylates Grb2-associated binder 1 upon hepatocyte growth factor stimulation. J Biol Chem. 2003;278(45):44075-82.

4. Garcia-Guzman M, Dolfi F, Zeh K, Vuori K. Met-induced JNK activation is mediated by the adapter protein Crk and correlates with the Gab1 - Crk signaling complex formation. Oncogene. 1999;18(54):7775-86.

5. Sakkab D, Lewitzky M, Posern G, Schaeper U, Sachs M, Birchmeier W, et al. Signaling of hepatocyte growth factor/scatter factor (HGF) to the small GTPase Rap1 via the large docking protein Gab1 and the adapter protein CRKL. J Biol Chem. 2000;275(15):10772-8.

6. Li N, Batzer A, Daly R, Yajnik V, Skolnik E, Chardin P, et al. Guanine-nucleotide-releasing factor hSos1 binds to Grb2 and links receptor tyrosine kinases to Ras signalling. Nature. 1993;363(6424):85-8.

7. Dolfi F, Garcia-Guzman M, Ojaniemi M, Nakamura H, Matsuda M, Vuori K. The adaptor protein Crk connects multiple cellular stimuli to the JNK signaling pathway. Proc Natl Acad Sci U S A. 1998;95(26):15394-9.

8. Rossman KL, Der CJ, Sondek J. GEF means go: turning on RHO GTPases with guanine nucleotide-exchange factors. Nat Rev Mol Cell Biol. 2005;6(2):167-80.

9. Ponzetto C, Bardelli A, Maina F, Longati P, Panayotou G, Dhand R, et al. A novel recognition motif for phosphatidylinositol 3-kinase binding mediates its association with the hepatocyte growth factor/scatter factor receptor. Mol Cell Biol. 1993;13(8):4600-8.

10. Vanhaesebroeck B, Leevers SJ, Panayotou G, Waterfield MD. Phosphoinositide 3-kinases: a conserved family of signal transducers. Trends Biochem Sci. 1997;22(7):267-72.

11. Tolias KF, Cantley LC. Pathways for phosphoinositide synthesis. Chem Phys Lipids. 1999;98(1-2):69-77.

12. Scheid MP, Woodgett JR. Unravelling the activation mechanisms of protein kinase B/Akt. FEBS Lett. 2003;546(1):108-12.

13. King AJ, Wireman RS, Hamilton M, Marshall MS. Phosphorylation site specificity of the Pak-mediated regulation of Raf-1 and cooperativity with Src. FEBS Lett. 2001;497(1):6-14.

14. Chen Z, Gibson TB, Robinson F, Silvestro L, Pearson G, Xu B, et al. MAP kinases. Chem Rev. 2001;101(8):2449-76.

15. Robinson MJ, Cobb MH. Mitogen-activated protein kinase pathways. Curr Opin Cell Biol. 1997;9(2):180-6.

16. Frödin M, Jensen CJ, Merienne K, Gammeltoft S. A phosphoserine-regulated docking site in the protein kinase RSK2 that recruits and activates PDK1. EMBO J. 2000;19(12):2924-34.

17. Puto LA, Pestonjamasp K, King CC, Bokoch GM. p21-activated kinase 1 (PAK1) interacts with the Grb2 adapter protein to couple to growth factor signaling. J Biol Chem. 2003;278(11):9388-93.

18. Edwards DC, Sanders LC, Bokoch GM, Gill GN. Activation of LIM-kinase by Pak1 couples Rac/Cdc42 GTPase signalling to actin cytoskeletal dynamics. Nat Cell Biol. 1999;1(5):253-9.

19. Montagner A, Yart A, Dance M, Perret B, Salles J-P, Raynal P. A novel role for Gab1 and SHP2 in epidermal growth factor-induced Ras activation. J Biol Chem. 2005;280(7):5350-60.

20. Cox AD, Der CJ. The dark side of Ras: regulation of apoptosis. Oncogene. 2003;22(56):8999-9006.

21. Rosário M, Birchmeier W. How to make tubes: signaling by the Met receptor tyrosine kinase. Trends Cell Biol. 2003;13(6):328-35.

22. Dance M, Montagner A, Salles J-P, Yart A, Raynal P. The molecular functions of Shp2 in the Ras/Mitogen-activated protein kinase (ERK1/2) pathway. Cell Signal. 2008;20(3):453-9.

23. Stork PJS. Does Rap1 deserve a bad Rap? Trends Biochem Sci. 2003;28(5):267-75.

24. Gao L, Feng Y, Bowers R, Becker-Hapak M, Gardner J, Council L, et al. Ras-associated protein-1 regulates extracellular signal-regulated kinase activation and migration in melanoma cells: two processes important to melanoma tumorigenesis and metastasis. Cancer Res. 2006;66(16):7880-8.

25. Douville E, Downward J. EGF induced SOS phosphorylation in PC12 cells involves P90 RSK-2. Oncogene. 1997;15(4):373-83.

26. Shin S-Y, Rath O, Choo S-M, Fee F, McFerran B, Kolch W, et al. Positive- and negative-feedback regulations coordinate the dynamic behavior of the Ras-Raf-MEK-ERK signal transduction pathway. J Cell Sci. 2009;122(Pt 3):425-35.

27. Yeung K, Seitz T, Li S, Janosch P, McFerran B, Kaiser C, et al. Suppression of Raf-1 kinase activity and MAP kinase signalling by RKIP. Nature. 1999;401(6749):173-7.

28. Dougherty MK, M\"uller Ju, Ritt DA, Zhou M, Zhou XZ, Copeland TD, et al. Regulation of Raf-1 by direct feedback phosphorylation. Mol Cell. 2005;17(2):215-24.

29. Sato S, Fujita N, Tsuruo T. Involvement of 3-phosphoinositide-dependent protein kinase-1 in the MEK/MAPK signal transduction pathway. J Biol Chem. 2004;279(32):33759-67.

30. Innocenti M, Frittoli E, Ponzanelli I, Falck JR, Brachmann SM, Fiore PPD, et al. Phosphoinositide 3-kinase activates Rac by entering in a complex with Eps8, Abi1, and Sos-1. J Cell Biol. 2003;160(1):17-23.

31. Innocenti M, Tenca P, Frittoli E, Faretta M, Tocchetti A, Fiore PPD, et al. Mechanisms through which Sos-1 coordinates the activation of Ras and Rac. J Cell Biol. 2002;156(1):125-36.

32. Eblen ST, Slack-Davis JK, Tarcsafalvi A, Parsons JT, Weber MJ, Catling AD. Mitogen-activated protein kinase feedback phosphorylation regulates MEK1 complex formation and activation during cellular adhesion. Mol Cell Biol. 2004;24(6):2308-17.

33. Higuchi M, Onishi K, Kikuchi C, Gotoh Y. Scaffolding function of PAK in the PDK1-Akt pathway. Nat Cell Biol. 2008;10(11):1356-64.

34. Yu CF, Roshan B, Liu ZX, Cantley LG. ERK regulates the hepatocyte growth factor-mediated interaction of Gab1 and the phosphatidylinositol 3-kinase. J Biol Chem. 2001;276(35):32552-8.

35. Downward J. Lipid-regulated kinases: some common themes at last. Science. 1998;279(5351):673-4.

36. Maroun CR, Holgado-Madruga M, Royal I, Naujokas MA, Fournier TM, Wong AJ, et al. The Gab1 PH domain is required for localization of Gab1 at sites of cell-cell contact and epithelial morphogenesis downstream from the met receptor tyrosine kinase. Mol Cell Biol. 1999;19(3):1784-99.

37. Mabuchi S, Ohmichi M, Kimura A, Hisamoto K, Hayakawa J, Nishio Y, et al. Inhibition of phosphorylation of BAD and Raf-1 by Akt sensitizes human ovarian cancer cells to paclitaxel. J Biol Chem. 2002;277(36):33490-500.

38. Zimmermann S, Moelling K. Phosphorylation and regulation of Raf by Akt (protein kinase B). Science. 1999;286(5445):1741-4.

39. Klamt S, Saez-Rodriguez J, Gilles ED (2007) Structural and functional analysis of cellular networks with CellNetAnalyzer. BMC systems biology 1: 2.
