# Supplementary material for: Disentangling the Complexity of HGF Signaling by Combining Qualitative and Quantitative Modeling
Source: PLoS Comput Biol. 2015 Apr 23;11(4):e1004192. doi: 10.1371/journal.pcbi.1004192 (PMC4427303; doi:10.1371/journal.pcbi.1004192)
Supplement: S1 Code — Pseudocodes for the selection of minimal model structures from the interaction graph master model. (PDF) [file pcbi.1004192.s020.pdf]

Here, we specify the pseudocodes for the selection of minimal model structures from the interaction graph master model. The user calls the function "SelectModels" (pseudocode this page), returning the building blocks of the selected minimal model structures. During the computation, two subroutines are called: "CNAcomputeDepMat", a *CellNetAnalyzer* API function [39] that computes the dependency matrix of a given model structure, and "DepMatCompare" (pseudocode next page), which compares the model predictions derived from the dependency matrix with the discretized experimental data.

Pseudocode of function SelectModels

```
Function BuildingBlocks ⟵ SelectModels (DataEarly,DataLate,Inhibitors,CoreModel,CandEdges)
inputs:
    DataEarly: for each measured protein, discretized fold change of different pairwise comparisons of experiments,
    early response
    DataLate: for each measured protein, discretized fold change of different pairwise comparisons of experiments,
    late response
    Inhibitors: for each pairwise comparison of experiments, applied inhibitor(s) making up the difference of the two
    experiments
    CoreModel: Interaction graph core model in CellNetAnalyzer format
    CandEdges: candidate edges
output:
    BuildingBlocks: building blocks of selected minimal model structures

/* compute dependency matrix of core model                                              */
DepMat ⟵ CNAcomputeDepMat (CoreModel);
call DepMatCompare (DataEarly,DataLate,Inhibitors,DepMat) returning Mismatch;
if Mismatch = 0 then
|   print 'Core model in accordance with data';
|   return;
end
BuildingBlocks ⟵ empty set;
for i from 1 to number of CandEdges do
|   [Combi_1 , . . . , Combi_K ] ⟵ all i-combinations of CandEdges;
|   for j from 1 to K do
|   |   if Combi_j is not superset of an element of BuildingBlocks then
|   |   |   /* compute dependency matrix of core model plus edges from Combi_j          */
|   |   |   Model ⟵ CoreModel plus edges from Combi_j;
|   |   |   DepMat ⟵ CNAcomputeDepMat (Model);
|   |   |   call DepMatCompare (DataEarly,DataLate,Inhibitors,DepMat) returning Mismatch;
|   |   |   if Mismatch = 0 then
|   |   |   |   append Combi_j to BuildingBlocks;
|   |   |   end
|   |   end
|   end
end
```

Pseudocode of function DepMatCompare

```
Function Mismatch ⟵ DepMatCompare (DataEarly,DataLate,Inhibitors,DepMat)
inputs:
    DataEarly: for each measured protein, discretized fold change of different pairwise comparisons of experiments,
    early response
    DataLate: for each measured protein, discretized fold change of different pairwise comparisons of experiments,
    late response
    Inhibitors: for each pairwise comparison of experiments, applied inhibitor(s) making up the difference of the two
    experiments
    DepMat: dependency matrix
output:
    Mismatch: number of mismatches between model predictions and discretized data

for i from 1 to number of measured species do
    for j from 1 to number of pairwise comparisons of experiments do
        if all Inhibitors of jth comparison are weak/strong activators for species i then /* this information
        is taken from DepMat                                                                              */
            ModelPredictionsEarly(i, j) ⟵ decrease;
        else if all Inhibitors of jth comparison are weak/strong inhibitors for species i then
            ModelPredictionsEarly(i, j) ⟵ increase;
        else if all Inhibitors of jth comparison are neutral factors for species i then
            ModelPredictionsEarly(i, j) ⟵ no change;
        else
            ModelPredictionsEarly(i, j) ⟵ increase, decrease, or no change;
        end
        if all Inhibitors of jth comparison are strong activators for species i then
            ModelPredictionsLate(i, j) ⟵ decrease;
        else if all Inhibitors of jth comparison are strong inhibitors for species i then
            ModelPredictionsLate(i, j) ⟵ increase;
        else if all Inhibitors of jth comparison are neutral factors for species i then
            ModelPredictionsLate(i, j) ⟵ no change;
        else
            ModelPredictionsLate(i, j) ⟵ increase, decrease, or no change;
        end
    end
end
Mismatch ⟵ count mismatches between DataEarly and ModelPredictionsEarly and DataLate and
            ModelPredictionsLate;
```
